# Memetic computing through bio-inspired heuristics integration with sequential quadratic programming for nonlinear systems arising in different physical models

**DOI:** 10.1186/s40064-016-3750-8

**Published:** 2016-12-01

**Authors:** Muhammad Asif Zahoor Raja, Adiqa Kausar Kiani, Azam Shehzad, Aneela Zameer

**Affiliations:** 1 Department of Electrical Engineering, COMSATS Institute of Information Technology, Attock Campus, Attock, Pakistan; 2 Department of Economics, Federal Urdu University of Arts Science and Technology, Islamabad, Pakistan; 3 Department of Mathematics, Preston University, Kohat, Islamabad Campus, Islamabad, Pakistan; 4 Department of Computer and Information Sciences, Pakistan Institute of Engineering and Applied Sciences (PIEAS), Nilore, Islamabad, 45650 Pakistan

**Keywords:** Nonlinear systems, Hybrid computing, Genetic algorithms, Sequential quadratic programming, Nonlinear equations

## Abstract

**Background:**

In this study, bio-inspired computing is exploited for solving system of nonlinear equations using variants of genetic algorithms (GAs) as a tool for global search method hybrid with sequential quadratic programming (SQP) for efficient local search. The fitness function is constructed by defining the error function for systems of nonlinear equations in mean square sense. The design parameters of mathematical models are trained by exploiting the competency of GAs and refinement are carried out by viable SQP algorithm.

**Results:**

Twelve versions of the memetic approach GA-SQP are designed by taking a different set of reproduction routines in the optimization process. Performance of proposed variants is evaluated on six numerical problems comprising of system of nonlinear equations arising in the interval arithmetic benchmark model, kinematics, neurophysiology, combustion and chemical equilibrium. Comparative studies of the proposed results in terms of accuracy, convergence and complexity are performed with the help of statistical performance indices to establish the worth of the schemes.

**Conclusions:**

Accuracy and convergence of the memetic computing GA-SQP is found better in each case of the simulation study and effectiveness of the scheme is further established through results of statistics based on different performance indices for accuracy and complexity.

## Background

### Introduction

Solving system of non-linear equations in particular based on large set are generally considered to be the most difficult and a challenging problem for the research community in the field of numerical computation. These systems arise frequently in a spectrum of applied mathematics and engineering applications including trajectory planning, kinematics, combustion theory and neurophysiology etc. (Grosan and Abraham 2008a, b; Morgan 1987). Currently, number of numerical methods have been developed to deal with nonlinear equations effectively, however, one of the simplest, oldest and widely used solvers for these problems is the Newton–Raphson method (NRM) (Ortega and Rheinboldt 1970; Kelley 2003). Similar to most of the numerical methods for solving system of non-linear equations, the performance of the NRM can be highly sensitive to the initial guess of the problem and generally fail with bad initial parameters. Therefore, normally, any global search methodology is used to determine the initial bias values which are then supplied to the NRM for solving viably the system of non-linear equations. Besides NRM, many other iterative methods for solving linear and nonlinear equations are reported in the literature with their own strengths, limitations and applicability domain on specific scenarios or environments. For instance, Kelley, Campbell, and Broyden’s classically provide different solvers for these equations (Kelley 1999; Campbell et al. 1996; Darvishi and Barati 2007). Moreover, the Jacobian-free Newton–Krylov method is applied broadly for non-linear equations arising in many applications in which an effective two sided bi-colouring method is used to get the lower triangular half of the sparse Jacobian matrix via automatic differentiation (Broyden 1971; Knoll and Keyes 2004; Saad and van der Vorst 2000). Recently, many researchers, including Jaffari and Gejji, Abbasbandy, Sharma et al., Vahidi et al. have given an updated version of methods to solve the nonlinear system of equation reliably and efficiently (Jafari and Gejji 2006; Abbasbandy 2005; Vahidi et al. 2012; Sharma and Guha 2013; Sharma and Gupta 2013, 2014; Sharma and Arora 2014).

Most of the existing literature available for solving nonlinear system of equation is based on iterative and recursive procedure, and working on these methods is usually dependent on values of initial guess or start point of the algorithms. On the other hand, these systems of equations have been used in modelling of many physical problems arising in a wide spectrum of fields (Morgan 1987; de Soares 2013). Therefore, design of numerical procedures that are accurate, reliable, robust, and efficient, has attracted the research community significantly. The aim of this study is to step further in this domain by exploring and exploiting the strength of soft computing framework (SCF) to determine the solution of systems of nonlinear equation without prior knowledge of biased initial guess or weights. The soft computing techniques based on genetic algorithms and swarming intelligence has been used extensively for different applications such as Van-der-Pol oscillatory systems (Khan et al. 2015), reliable feature selection for Arabic text summarization (Al-Zahrani et al. 2015), effective navigation of mobile robot in unknown environment(Algabri et al. 2014), robust feature selection and classification (Nekkaa and Boughaci 2015), fuel ignition model in combustion theory (Raja 2014), change detection mechanism in synthetic aperture radar images (Li et al. 2015), optimization of multirate quadrature mirror filter bank (Baicher 2012), integrated process planning and scheduling problems (Li et al. 2014), thin film flow of third grade fluids (Raja et al. 2014), Troesch’s problem (Raja 2014), second order system of boundary value problems (Arqub and Abo-Hammour 2014; Abu-Arqub et al. 2014), prediction of linear dynamical systems (Abo-Hammour et al. 2013), Jeffery-Hamel Flow in the presence of high magnetic field (Raja and Samar 2014), Painlevé equations (Raja et al. 2015), modeling of electrical conducting solids (Raja et al. 2016), nanofludics problems (Raja et al. 2016), Riccati fractional differential equations (FrDEs) (Raja et al. 2015), real time cross layer optimization (Elias et al. 2012) and Bagley-Torvik FrDEs (Raja et al. 2011). These are the motivating factors for the authors to explore in this domain. The objective of this study is to design memetic evolutionary techniques based on effective global search and efficient local search methodologies and then apply the proposed SCF for an accurate, effective and reliable solution of system of nonlinear equations.

The rest of the organization of the paper is as follows. In “Methods” section, proposed design methodology is presented for the solutions of nonlinear system of equation by formulation of fitness functions, stepwise working criteria and its learning mechanism. In “Results and discussion” section, the results of numerical experimentations of proposed schemes are presented for six benchmark problems, including application arising in combustion theory, neurophysiology and Kinetic modelling etc. along the comparison of the results in term of performance operators. In “Comparative studies” section, results of the proposed algorithms are compared using statistical performance indicators for both accuracy and complexity. Concluding remarks as well as future research directions are given in the last section.

## Methods

In this section, design methodology is presented for finding the solution of a system of nonlinear equations. Our aim is to provide a platform for optimization of variables for the given system in order to find the accurate and precise solution. Genetic algorithms (GAs) is an optimization tool which can be used effectively for finding the solution of a given system of nonlinear equation without using the initial guess.

### Formulation of fitness function

Generally, system of nonlinear equation is expressed as:

1 $${\mathbf{F}}(t) = 0,$$

or

2 $${\mathbf{F}}(t) = \left\{ {\begin{array}{*{20}l} {f_{1} (t_{1} ,t_{2} ,t_{3} , \ldots ,t_{n} ) = 0,} \hfill \\ {f_{2} (t_{1} ,t_{2} ,t_{3} , \ldots ,t_{n} ) = 0,} \hfill \\ \vdots \hfill \\ {f_{n} (t_{1} ,t_{2} ,t_{3} , \ldots ,t_{n} ) = 0} \hfill \\ \end{array} } \right..$$

To find out the precise solution of (1), the first step is to formulate the fitness or objective function on the basis of absolute value of the function or mean square error as:

3 $${\text{Minimize}}\,\,\left\{ {\,\varepsilon = \left| {{\mathbf{F}}(t)} \right|\,\,\,\,\,{\text{or}}\,\,\,\, = \left( {{\mathbf{F}}(t)} \right)^{2} } \right..$$

Fitness function (3) depends upon bounded or unbounded constraints on variables and these variables are used to define the given system of nonlinear equations.

The next step is to optimize the formulated fitness function (3) by using reliable optimization mechanisms such that for $\varepsilon \to 0$ then ${\mathbf{F}}(t) \to 0$. Eventually the correspondingly values of the vector $t = (t_{1} ,t_{2} ,t_{3} , \ldots ,t_{n} )$ are the solution set for the given system of nonlinear equation.

### Learning methodology

From the last few decades’ mathematicians and researchers made serious efforts to produce quality of initial guesses to find the optimal final solutions. This was really tough, especially when the number of variables involving in the system of nonlinear equations exceeds a specific level. Genetic algorithms (GAs) are basically the modelling of the phenomenon of natural evolution (Miettinen 1999). The effectiveness and systematic operation of GA’s depends upon not only the selection of different constitutional operators, but also on the settings of algorithm, variable parameters and the design constraints.

GAs is considered to be one of the best optimization algorithm as compared to the others because of their multi-dimensionally operations to produce most feasible solutions. GAs are also effective for the problems for which the construction of fitness function is much complex (Johnson et al. 2014; Kociecki and Adeli 2014). In our daily life, most problems have a very large solution space, which is generally not dealt by the ordinary algorithms while GAs deals these situations efficiently and correctly. GAs is considered to be the most viable and accurate method for finding the numerical approximate solutions of the given system of nonlinear equations by determining the best fit from the extensive range of search space. In the present study, the memetic computing approach is developed based on variants of GAs (Raja et al. 2015) hybrid with sequential quadratic programming (SQP) technique to obtain unknown design variables of nonlinear system given in Eq. (1).

**Fig. 1:**
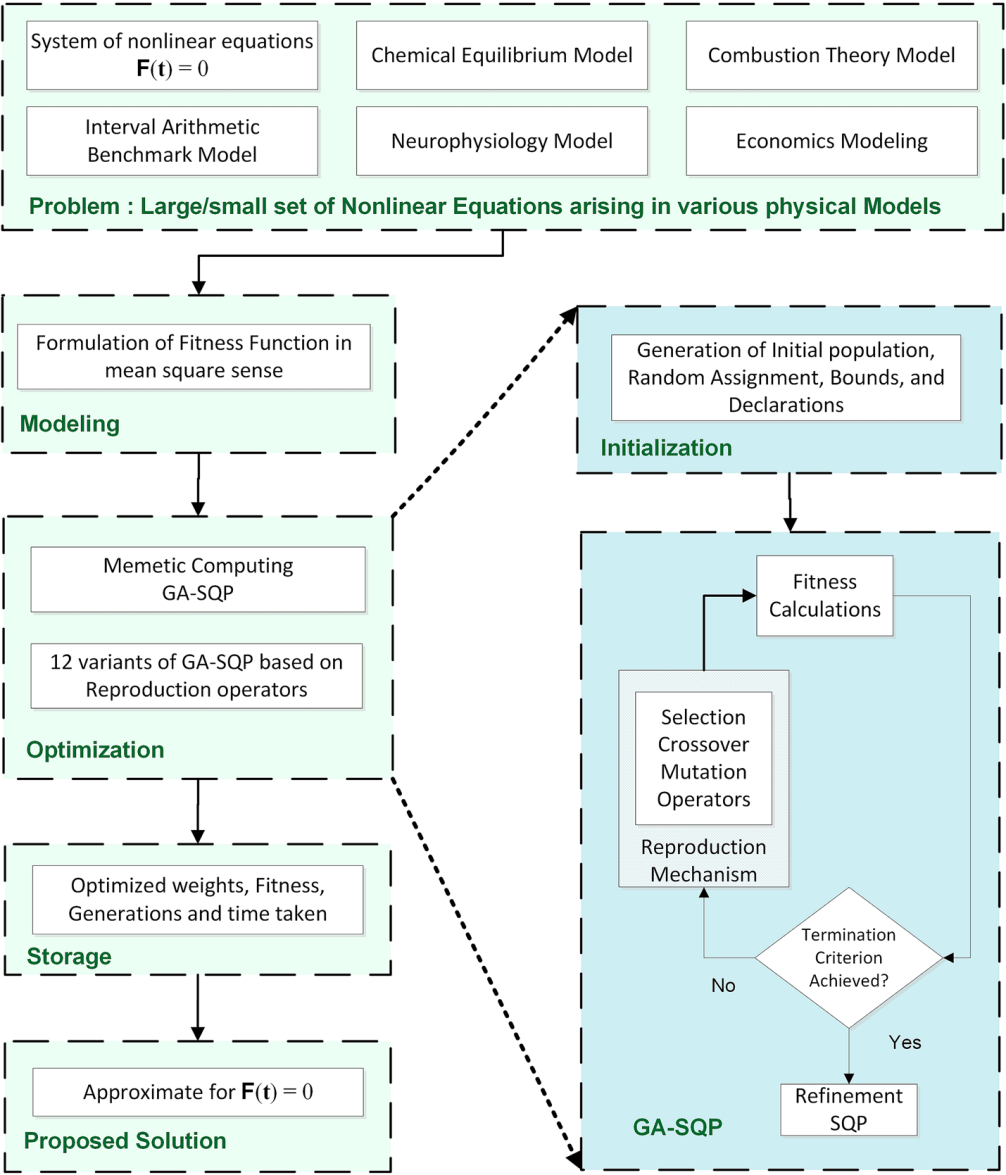
Graphical view of proposed design methodology

The different computing approaches based on variants of GA hybrid with SQP are evaluated for finding the solution of systems of nonlinear equations. The proposed hybrid computing schemes are listed in Table 1, while procedural overview is given graphically in Fig. 1. Necessary details of procedural steps for hybrid computing algorithms are given below:

Step 1: *Initialization* Initialize the chromosome with number of elements equal to the number of variable in nonlinear system of equations as:
  $$c = (t_{1} ,t_{2} ,t_{3} , \ldots ,t_{n} ),$$
  where *n* represents variables in nonlinear system. Set of chromosome represents an initial population and it is given mathematically as:
  $$P = (c_{1} ,c_{2} ,c_{3} , \ldots ,c_{m} ),$$
  here *m* is the total number of chromosomes in the population *P*.
  Initial assignments and declarations of GAs program and are set using MATLAB built-in functions of the optimization toolbox based on ‘ga’ and ‘gaoptimset’ routines. The fix parameter settings of all twelve variants of GAs are used such as 200 generations. The choice of these settings is made with care, after a lot of experimentation, and experience of operating optimization solvers.
Step 2: *Fitness calculation* Evaluate the fitness values of each individual or chromosome of the population using a problem specific fitness function as defined in (3).
Step 3: *Termination criteria* Terminate the updating process of the algorithm, if any of the following predefined conditions are fulfilled.
  - Fitness *ε* values are less than or equal to 10^−35^.
  - Generations, i.e., 400 times step increment are made in the program execution.
  - Limited values of any of the functions, tolerance (TolFun), constraints tolerance (TolCon) and stall generation limit (StallGenLimit) is achieved.
  Go to step 5 in case of termination conditions are satisfied.
Step 4: *Reproduction* Create the next generation of each variant of GAs based on a different set of combination for the reproduction mechanism using selection, crossover, and mutation routines as listed in Table 1. Go to step 2
Step 5: *Hybridization* Global best individual of GAs variants is given to a local search method based on SQP algorithm, i.e., the memetic computing approach of learning, for further refinements in the results. SQP algorithm is implemented by invoking ‘fmincon’ function of MATLAB optimization toolbox for constraint problems as per following procedure:
  - *Initialization* Initial weights or start point of SQP algorithm is the global best chromosomes of GAs variants. The bounds, declarations and initial parameters are given in ‘*optimset*’ function such as number of iteration 500.
  - *Fitness calculation* Calculate the fitness value using the Eq. (3).
  - *Termination criteria* Terminate the cyclic process of updating in variables if any of the following predefine conditions fulfilled.
    Total number of Iterations/cycles are executed.
    Limited values for any TolFun, maximum function evaluations (MaxFunEvals), X-tolerance (TolX), and TolCon are achieved as given in ‘*optimset*’ function.
    Go to the step 6 in case of termination conditions satisfied.
  - *Updating of variables*: Updating of weights is made on each step increment as per SQP procedure and continues from step 5(b).
Step 6: *Storage* Store the values of the weights, fitness, generations, MaxFunEvals and time taken for this run in case of all twelve hybrid schemes based on GA-SQP.
Step 7: *Statistical analysis* Repeat the procedure for a sufficient large number of times from step 1 to step 6 to generate large data set for reliable and effective analysis of the performance of the algorithms.

**Table 1:** Proposed algorithms for system of nonlinear equations

Memetic algorithms	Global search operators for gas			Local search method
	Selection	Crossover	Mutations	
GA-SQP-1	Stochastic uniform	Heuristic	Adaptive feasible	SQP
GA-SQP-2	Stochastic uniform	Heuristic	Gaussian	SQP
GA-SQP-3	Stochastic uniform	Arithmetic	Adaptive feasible	SQP
GA-SQP-4	Stochastic uniform	Arithmetic	Gaussian	SQP
GA-SQP-5	Reminder	Heuristic	Adaptive feasible	SQP
GA-SQP-6	Reminder	Heuristic	Gaussian	SQP
GA-SQP-7	Reminder	Arithmetic	Adaptive feasible	SQP
GA-SQP-8	Reminder	Arithmetic	Gaussian	SQP
GA-SQP-9	Roulette	Heuristic	Adaptive feasible	SQP
GA-SQP-10	Roulette	Heuristic	Gaussian	SQP
GA-SQP-11	Roulette	Arithmetic	Adaptive feasible	SQP
GA-SQP-12	Roulette	Arithmetic	Gaussian	SQP

## Results and discussion

In this section, results of proposed schemes for solving system of nonlinear equations are presented. Six different problems are taken for the study and proposed methods base on twelve variants of memetic computing using GAs and SQP algorithms are applied for these equations. Six different models of system of nonlinear equation are taken for numerical experimentation to check the effectiveness of the proposed design schemes. These models with governing mathematical relations are described in six problems.

### Problem 1: generic nonlinear system of equations

In this case, a generic system of nonlinear equations based on four equations with four unknowns is taken and it is represented by the following mathematical relations (Grosan and Abraham 2008b)

4 $$\left\{ {\begin{array}{*{20}l} {f_{1} ({\mathbf{t}}) = f_{1} (t_{1} ,\,t_{2} ,\,t_{3} ,\,t_{4} ) = t_{1}^{2} + 2t_{2}^{2} + \cos (t_{3} ) - t_{4}^{2} = 0} \hfill \\ {f_{2} ({\mathbf{t}}) = f_{2} (t_{1} ,\,t_{2} ,\,t_{3} ,\,t_{4} ) = 3t_{1}^{2} + t_{2}^{2} + \sin^{2} (t_{3} ) - t_{4}^{2} = 0} \hfill \\ {f_{3} ({\mathbf{t}}) = f_{3} (t_{1} ,\,t_{2} ,\,t_{3} ,\,t_{4} ) = - 2t_{1}^{2} - t_{2}^{2} - \cos (t_{3} ) + t_{4}^{2} = 0} \hfill \\ {f_{4} ({\mathbf{t}}) = f_{4} (t_{1} ,\,t_{2} ,\,t_{3} ,\,t_{4} ) = - t_{1}^{2} - 2t_{2}^{2} \, - \cos^{2} (t_{3} ) + t_{4}^{2} = 0} \hfill \\ \end{array} } \right..$$

The exact solution for system (4) is (1, −1, 0, 2) for (*t*_1_, *t*_2_, *t*_3_, *t*_4_), respectively, and these solutions are used to verify the applicability of the proposed methods. The design approaches are applied to solve the system (4) as per procedure and settings given in “Results and discussion” section, however, the fitness function formulated in this case is given as:

5 $$\varepsilon = \frac{1}{4}\left( {\begin{array}{*{20}l} {\left( {t_{1}^{2} + 2t_{2}^{2} + \cos (t_{3} ) - t_{4}^{2} } \right)^{2} + \left( {3t_{1}^{2} + t_{2}^{2} + \sin^{2} (t_{3} ) - t_{4}^{2} } \right)^{2} } \hfill \\ {\quad +\, \left( { - 2t_{1}^{2} - t_{2}^{2} - \cos (t_{3} ) + t_{4}^{2} } \right)^{2} + \left( { - t_{1}^{2} - 2t_{2}^{2} \, - \cos^{2} (t_{3} ) + t_{4}^{2} } \right)^{2} } \hfill \\ \end{array} } \right).$$

**Fig. 2:**
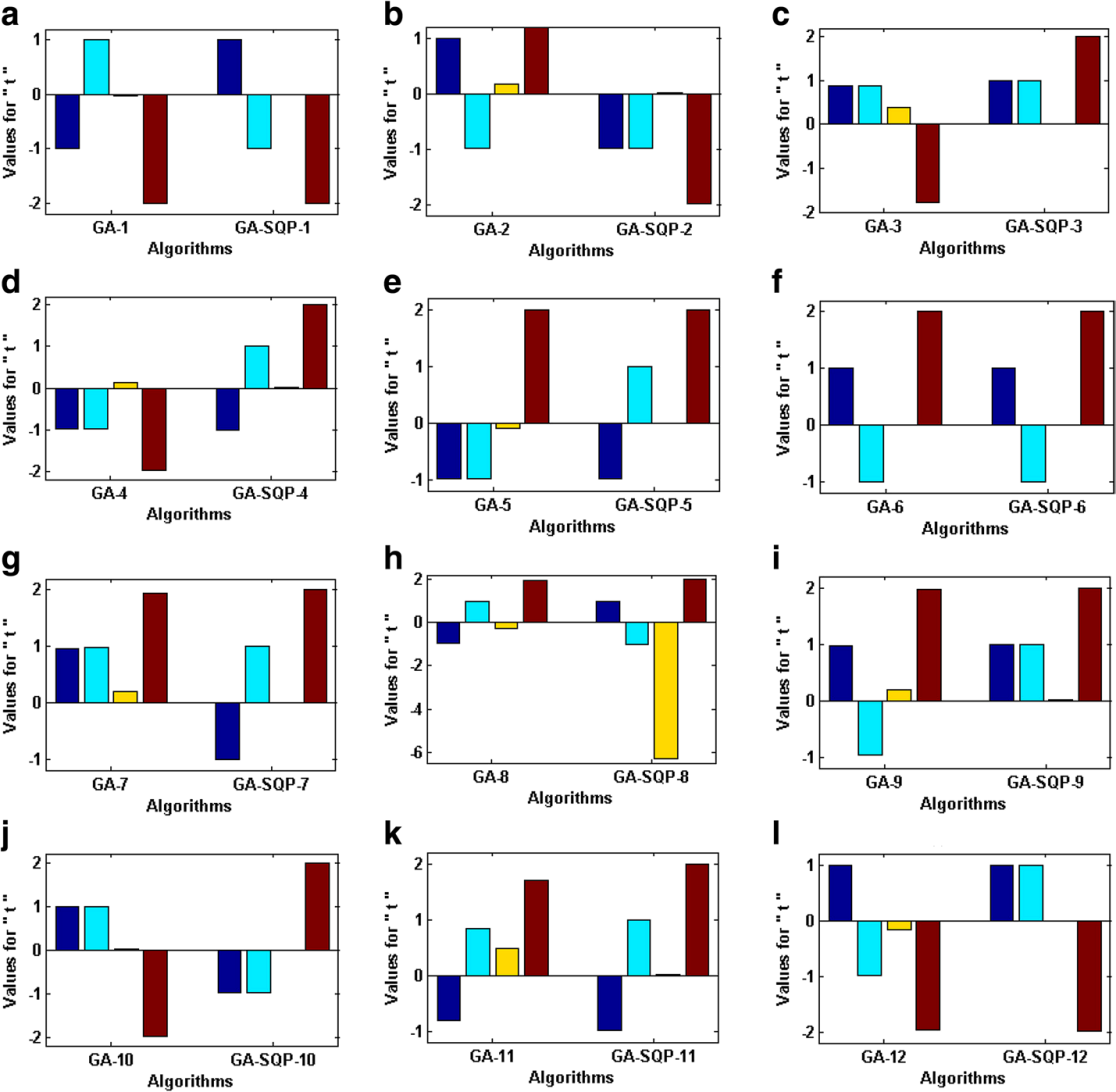
Trained parameters of GA and GA-SQP algorithms in cases of problem 1, while the figures (**a**–**l**), represent the results of variant *1*–*12* of proposed methodology, respectively

Optimization of fitness function (5) is carried out with twelve variants of memetic computing algorithms and one set of solution obtained by GAs and GA-SQP algorithm is shown in Fig. 2 for each variant. While these adaptive parameters along with their fitness values are tabulated in Table 2.

**Table 2:** Comparison of trained parameters along with their fitness for GA and GA-SQP algorithms in case of problem 1

Method	Proposed solutions				*ɛ*
	*t*_1_	*t*_2_	*t*_3_	*t*_4_	
GA-1	−0.999404287	0.999479968	−0.027480501	−1.999042387	1.8262E−08
GA-2	0.979068695	−0.981748424	0.163179035	1.966420060	2.1943E−05
GA-3	0.868284707	0.877541519	0.392426227	−1.784981013	7.0007E−04
GA-4	−0.987183026	−0.987817444	0.124553341	−1.978604103	8.6898E−06
GA-5	−0.990822818	−0.991856983	−0.106387112	1.985169394	4.1780E−06
GA-6	0.999913716	−0.999917219	−0.010038843	1.999856079	3.8862E−10
GA-7	0.966617064	0.970313641	0.198841379	1.946148641	5.2871E−05
GA-8	−0.950190958	0.965065042	−0.277206136	1.925861774	2.0243E−04
GA-9	0.973598309	−0.976106840	0.178440757	1.956961516	3.3514E−05
GA-10	0.999854603	0.999934568	0.017078799	−1.999804148	5.4235E−09
GA-11	−0.812150872	0.832453398	0.475826424	1.699196146	1.2769E−03
GA-12	0.980516502	−0.984157549	−0.162937302	−1.969458167	2.1478E−05
GA-SQP-1	0.999999973	−0.999999974	0.000178418	−1.999999955	3.8017E−17
GA-SQP-2	−0.999999973	−0.999999974	0.000178410	−1.999999955	3.8011E−17
GA-SQP-3	0.999999973	0.999999974	0.000178411	1.999999955	3.8012E−17
GA-SQP-4	−0.999999973	0.999999974	0.000178416	1.999999955	3.8015E−17
GA-SQP-5	−0.999999973	0.999999974	−0.000178417	1.999999955	3.8016E−17
GA-SQP-6	0.999999973	−0.999999974	−0.000178410	1.999999955	3.8011E−17
GA-SQP-7	−0.999999973	0.999999974	−0.000178410	1.999999955	3.8011E−17
GA-SQP-8	0.999999975	−0.999999977	−6.283010586	1.999999959	3.3944E−17
GA-SQP-9	0.999999973	0.999999974	0.000178410	1.999999955	3.8012E−17
GA-SQP-10	−0.999999973	−0.999999974	−0.000178408	1.999999955	3.8010E−17
GA-SQP-11	−0.999999973	0.999999974	0.000178430	1.999999955	3.8025E−17
GA-SQP-12	0.999999973	0.999999974	−0.000178417	−1.999999955	3.8016E−17

The optimized variables given in Table 2 and Fig. 2 are used Eq. (4) in order to determine the values of $f_{1} ({\mathbf{t}})$, $f_{2} ({\mathbf{t}})$, $f_{3} ({\mathbf{t}})$ and, $f_{4} ({\mathbf{t}})$ and results are given in Table 3. The smaller is the values of fitness then better is the performance of the algorithm

**Table 3:** Comparison of the performance on the basis of absolute values of constitutional equations of problem 1

Method	Absolute values				Method	Absolute values			
	$f_{1} ({\mathbf{t}})$	$f_{2} ({\mathbf{t}})$	$f_{3} ({\mathbf{t}})$	$f_{4} ({\mathbf{t}})$		$f_{1} ({\mathbf{t}})$	$f_{2} ({\mathbf{t}})$	$f_{3} ({\mathbf{t}})$	$f_{4} ({\mathbf{t}})$
GA-1	1.81E−04	2.85E−05	3.00E−05	1.96E−04	GA-SQP-1	7.48E−09	2.68E−09	4.22E−09	8.44E−09
GA-2	6.14E−03	8.59E−04	8.89E−04	6.96E−03	GA-SQP-2	7.48E−09	2.68E−09	4.22E−09	8.43E−09
GA-3	3.19E−02	8.07E−03	1.57E−02	3.83E−02	GA-SQP-3	7.48E−09	2.68E−09	4.22E−09	8.43E−09
GA-4	3.48E−03	6.64E−05	2.22E−03	4.21E−03	GA-SQP-4	7.48E−09	2.68E−09	4.22E−09	8.43E−09
GA-5	2.74E−03	6.52E−04	6.89E−04	2.88E−03	GA-SQP-5	7.48E−09	2.68E−09	4.22E−09	8.43E−09
GA-6	2.16E−05	6.80E−06	1.46E−05	2.88E−05	GA-SQP-6	7.48E−09	2.68E−09	4.22E−09	8.43E−09
GA-7	1.02E−02	3.92E−03	3.01E−03	9.15E−03	GA-SQP-7	7.48E−09	2.68E−09	4.22E−09	8.43E−09
GA-8	1.84E−02	5.89E−03	1.00E−02	1.83E−02	GA-SQP-8	5.42E−09	3.58E−10	3.06E−09	9.85E−09
GA-9	7.89E−03	1.73E−03	3.00E−03	7.74E−03	GA-SQP-9	7.48E−09	2.68E−09	4.22E−09	8.43E−09
GA-10	8.50E−05	7.18E−05	7.49E−05	6.08E−05	GA-SQP-10	7.48E−09	2.68E−09	4.22E−09	8.43E−09
GA-11	4.72E−02	5.69E−03	1.38E−02	5.16E−02	GA-SQP-11	7.48E−09	2.68E−09	4.22E−09	8.44E−09
GA -12	6.53E−03	3.53E−04	6.19E−04	6.54E−03	GA-SQP-12	7.48E−09	2.68E−09	4.22E−09	8.43E−09

It is seen from Table 2 that the values of fitness *ε* for variants of GAs are around 10^−04^ to 10^−10^ while these values for the hybrid approach GA-SQP variants are around 10^−17^. Moreover, it is seen from Table 3 that the values of, $f_{i} ({\mathbf{t}})$, *i* = 1–4, for GAs and GA-SQP algorithms lie 10^−02^ to 10^−05^, and 10^−09^, respectively. There is no noticeable difference between the performances of the memetic computing approaches; however, the best results are obtained with GA-SQP-8 algorithm. It is observed that generally highly accurate results are determined by memetic computing techniques than variants of GAs.

### Problem 2: interval arithmetic benchmark model

In this case, the performance of the proposed methods is evaluated by taking a renewed problem of nonlinear systems named as an interval arithmetic benchmark model (IABM). The governing mathematical relations for IABM is given in the form of following nonlinear system of equations (Grosan and Abraham 2008b; Van Hentenryck et al. 1997; Hong and Stahl 1994):

6 $$\left\{ {\begin{array}{*{20}l} {f_{1} ({\mathbf{t}}) = t_{1} - 0.25428722 - 0.18324757t_{4} t_{3} t_{9} = 0} \hfill \\ {f_{2} ({\mathbf{t}}) = t_{2} - 0.37842197 - 0.16275449t_{1} t_{10} t_{6} = 0} \hfill \\ {f_{3} ({\mathbf{t}}) = t_{3} - 0.27162577 - 0.16955071t_{1} t_{2} t_{10} = 0} \hfill \\ {f_{4} ({\mathbf{t}}) = t_{4} - 0.19807914 - 0.15585316t_{7} t_{1} t_{6} = 0} \hfill \\ {f_{5} ({\mathbf{t}}) = t_{5} - 0.44166728 - 0.19950920t_{7} t_{6} t_{3} = 0} \hfill \\ {f_{6} ({\mathbf{t}}) = t_{6} - 0.14654113 - 0.18922793t_{8} t_{5} t_{10} = 0} \hfill \\ {f_{7} ({\mathbf{t}}) = t_{7} - 0.42937161 - 0.21180486t_{2} t_{5} t_{8} = 0} \hfill \\ {f_{8} ({\mathbf{t}}) = t_{8} - 0.07056438 - 0.17081208t_{1} t_{7} t_{6} = 0} \hfill \\ {f_{9} ({\mathbf{t}}) = t_{9} - 0.34504906 - 0.19612740t_{10} t_{6} t_{8} = 0} \hfill \\ {f_{10} ({\mathbf{t}}) = t_{10} - 0.42651102 - 0.21466544t_{4} t_{8} t_{1} = 0} \hfill \\ \end{array} } \right.,$$

where ${\mathbf{t}} = \left( {t_{1} , \, t_{2} , \ldots ,t_{10} } \right)$. The design memetic computing approaches are applied to solve the system (5) on a similar pattern as adopted in last problem; however, the fitness function formulated in this case is given as follows:

7 $$\varepsilon = \frac{1}{10}\left( {\left( {f_{1} ({\mathbf{t}})} \right)^{2} + \left( {f_{2} ({\mathbf{t}})} \right)^{2} + \left( {f_{3} ({\mathbf{t}})} \right)^{2} + \cdots \left( {f_{10} ({\mathbf{t}})} \right)^{2} } \right)$$

**Fig. 3:**
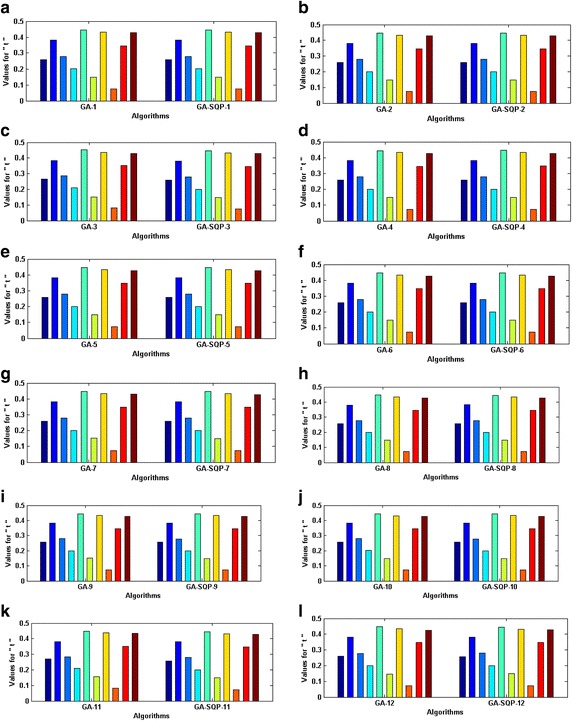
Trained parameters of GA and GA-SQP algorithms in case of problem *2*, while the figures (**a**–**l**), represent the results of variant *1*–*12* of proposed methodology, respectively

Optimization of fitness function (7) is carried out with the proposed scheme and results are shown graphically in Fig. 3 for variants of GAs and GA-SQP algorithms. The absolute values of constitutional equations are calculated and results are tabulated in Table 4 for each variant. It is seen that the maximum values of $f_{i} ({\mathbf{t}})$, *i* = 1–10, for GA and GA-SQP algorithms are 1.13 × 10^−02^ and 1.46 × 10^−16^, respectively. It is observed that generally the hybrid computing approaches give the results with higher precision from the rest.

**Table 4:** Comparison of trained parameters along with their fitness for GA and GA-SQP algorithms in case of problem 2

Method	Absolute values									
	$f_{1} ({\mathbf{t}})$	$f_{2} ({\mathbf{t}})$	$f_{3} ({\mathbf{t}})$	$f_{4} ({\mathbf{t}})$	$f_{5} ({\mathbf{t}})$	$f_{6} ({\mathbf{t}})$	$f_{7} ({\mathbf{t}})$	$f_{8} ({\mathbf{t}})$	$f_{9} ({\mathbf{t}})$	$f_{10} ({\mathbf{t}})$
GA-1	1.95E−04	8.59E−05	8.04E−05	6.73E−05	2.14E−06	1.99E−04	2.03E−04	5.08E−05	2.38E−05	1.28E−04
GA-2	1.11E−04	6.11E−05	1.45E−04	1.64E−05	1.59E−04	3.00E−04	9.51E−05	1.51E−04	1.06E−04	3.37E−04
GA-3	7.70E−03	2.72E−03	7.92E−03	9.06E−03	8.95E−03	8.62E−04	3.63E−03	7.65E−03	6.87E−03	2.55E−03
GA-4	1.16E−03	8.85E−04	7.90E−04	5.93E−04	2.00E−03	8.81E−04	2.61E−04	4.13E−04	8.22E−05	1.26E−03
GA-5	6.24E−05	1.51E−06	1.10E−04	1.18E−04	3.31E−05	8.62E−06	1.83E−05	3.80E−05	9.69E−05	3.09E−05
GA-6	7.95E−05	1.03E−04	6.85E−05	1.35E−04	4.90E−05	2.66E−04	2.10E−04	2.02E−04	2.03E−04	8.51E−05
GA-7	1.15E−03	1.79E−03	1.38E−03	2.72E−04	2.27E−03	3.74E−03	2.23E−03	7.52E−04	6.01E−04	2.69E−03
GA-8	1.33E−04	4.64E−04	1.22E−04	8.29E−05	9.25E−04	5.37E−05	2.72E−05	3.39E−04	7.71E−04	7.23E−04
GA-9	4.50E−04	3.23E−05	3.72E−04	3.20E−04	8.02E−05	6.25E−04	1.68E−04	2.28E−04	5.79E−05	3.10E−04
GA-10	4.43E−04	1.85E−05	4.26E−04	7.06E−05	4.04E−05	2.16E−04	1.50E−04	2.12E−04	5.02E−04	5.50E−04
GA-11	1.13E−02	1.90E−03	2.95E−03	1.00E−02	2.57E−03	6.58E−03	4.03E−03	7.04E−03	4.98E−03	7.41E−03
GA-12	1.09E−03	7.57E−04	1.41E−03	1.54E−04	1.86E−03	1.98E−03	1.13E−03	4.83E−04	1.59E−04	4.41E−03
GA-SQP-1	3.51E−17	9.63E−17	8.33E−17	1.69E−17	2.21E−17	1.09E−16	5.20E−18	3.08E−17	1.52E−16	2.47E−17
GA-SQP-2	1.46E−16	1.65E−17	2.69E−17	1.78E−17	1.44E−16	8.24E−17	5.07E−17	3.69E−17	4.24E−17	2.58E−17
GA-SQP-3	9.06E−17	7.33E−17	7.98E−17	8.24E−18	1.32E−16	2.60E−18	5.64E−17	9.02E−17	4.36E−17	2.63E−17
GA-SQP-4	2.39E−17	1.65E−17	8.76E−17	8.67E−18	3.73E−17	3.17E−17	6.29E−17	6.11E−17	1.25E−16	7.98E−17
GA-SQP-5	8.89E−17	3.73E−17	2.69E−17	6.42E−17	3.51E−17	2.65E−17	5.29E−17	6.33E−17	9.89E−17	2.92E−17
GA-SQP-6	3.34E−17	7.16E−17	8.07E−17	3.77E−17	2.34E−17	8.24E−17	1.16E−16	9.11E−18	6.90E−17	3.01E−17
GA-SQP-7	3.43E−17	1.65E−17	2.60E−17	3.73E−17	2.17E−17	5.46E−17	6.07E−17	4.77E−18	4.23E−17	8.58E−17
GA-SQP-8	3.47E−17	1.69E−17	7.98E−17	6.42E−17	2.13E−17	4.34E−19	1.15E−16	2.17E−17	9.82E−17	2.57E−17
GA-SQP-9	3.47E−17	1.65E−17	3.21E−17	4.68E−17	9.11E−17	2.60E−18	1.17E−16	6.07E−18	4.23E−17	8.03E−17
GA-SQP-10	2.21E−17	1.69E−17	1.36E−16	6.64E−17	3.25E−17	8.67E−19	5.25E−17	5.07E−17	6.79E−17	2.96E−17
GA-SQP-11	2.13E−17	9.45E−17	2.78E−17	4.60E−17	8.98E−17	2.60E−17	6.07E−17	1.86E−17	6.87E−17	8.65E−17
GA-SQP-12	1.32E−16	1.47E−17	2.08E−17	4.38E−17	3.38E−17	2.95E−17	1.73E−18	9.41E−17	1.20E−17	3.01E−17

### Problem 3: chemical equilibrium applications

Consider another potential example of a system of nonlinear equations arises in chemical equilibrium applications (CEA) (Grosan and Abraham 2008b; Meintjes and Morgan 1990) to evaluate the performance of the proposed schemes. Mathematical model of CEA is given below:

8 $$\left\{ {\begin{array}{*{20}l} {f_{1} ({\mathbf{t}}) = t_{1} t_{2} + t_{1} - 3t_{5} = 0} \hfill \\ {f_{2} ({\mathbf{t}}) = 2t_{1} t_{2} + t_{1} + t_{2} t_{3}^{2} + c{}_{8}t_{2} - ct_{5} + 2c_{10} t_{2}^{2} + c_{7} t_{2} t_{3} + c_{9} t_{2} t_{4} = 0} \hfill \\ {f_{3} ({\mathbf{t}}) = 2t_{2} t_{3}^{2} + 2c_{5} t_{3}^{2} - 8t_{5} + c_{6} t_{3} + c_{7} t_{2} t_{3} = 0} \hfill \\ {f_{4} ({\mathbf{t}}) = c_{9} t_{2} t_{4} + 2t_{4}^{2} - 4ct_{5} = 0} \hfill \\ {f_{5} ({\mathbf{t}}) = t_{1} (t_{2} + 1) + c_{10} t_{2}^{2} + t_{2} t_{3}^{2} + c_{8} t_{2} + c_{5} t_{3}^{2} + t_{4}^{2} - 1 + c_{6} t_{3} + c_{7} t_{2} t_{3} + c_{9} t_{2} t_{4} = 0} \hfill \\ \end{array} } \right.,$$

where $c = 10,$ $\,c_{5} = 0.193,$ $\,c_{6} = 0.000410,\,$ $c_{7} = 0.000545,$ $\,c_{8} = 0.000000449,$ $c_{9} = 0.0000340,$ and $c_{10} = 0.000000961$. The designed computing approaches are applied to solve the system (8) on a similar pattern as adopted in last problems, but the fitness function is formulated for this case as:

9 $$\varepsilon = \frac{1}{5}\left( {\left( {f_{1} ({\mathbf{t}})} \right)^{2} + \left( {f_{2} ({\mathbf{t}})} \right)^{2} + \left( {f_{3} ({\mathbf{t}})} \right)^{2} + (f_{4} ({\mathbf{t}}))^{2} + \left( {f_{5} ({\mathbf{t}})} \right)^{2} } \right)$$

An optimization problem based on a fitness function (9) is solved with twelve variants of memetic computing. The trained design variables are given in Table 5. These parameters are used to calculate the values of functions $f_{1} ({\mathbf{t}})$, $f_{2} ({\mathbf{t}})$, $f_{3} ({\mathbf{t}})$, $f_{4} ({\mathbf{t}})$ and $f_{5} ({\mathbf{t}})$. The results are given in Table 6. It is seen from Table 5 that the values of fitness *ε* for variants of GAs are of the order 10^−04^ to 10^−05^ while these values for variants of hybrid approach GA-SQPs are around 10^−17^. Additionally, it is seen from Table 6 that the values of $f_{i} ({\mathbf{t}})$, *i* = 1–5, for GA and GA-SQP algorithms lie around 10^−02^ to 10^−06^, and 10^−10^, respectively. It is observed that the best results are obtained with GA-SQP-8 algorithm from the rest.

**Table 5:** Comparison of trained parameters along with their fitness for GA and GA-SQP algorithms in case of problem 3

Method	Proposed solutions					*ɛ*
	*t*_1_	*t*_2_	*t*_3_	*t*_4_	*t*_5_	
GA-1	0.03228963	2.85389240	0.21989919	−0.85284380	0.03635484	6.6071E−05
GA-2	0.03754135	2.38952055	0.23779156	0.85132675	0.03623233	9.0098E−05
GA-3	0.06308618	1.18624138	0.32683618	0.84488974	0.03568812	2.6746E−04
GA-4	0.05434276	1.46521255	0.29761121	−0.84773318	0.03592740	1.9541E−04
GA-5	0.02704748	3.51739016	0.19943025	0.85423864	0.03648805	4.5340E−05
GA-6	0.02302472	4.24026955	0.18171406	0.85266824	0.03634051	3.8101E−05
GA-7	0.06294020	1.18814861	0.32687402	−0.84344415	0.03555521	2.6983E−04
GA-8	0.06329114	1.14885683	−0.33415631	−0.84616131	0.03575514	2.8793E−04
GA-9	0.03477842	2.60523708	0.22955001	0.85209604	0.03630524	7.6619E−05
GA-10	0.03333412	2.61711165	0.23398167	0.85554477	0.03670055	1.2481E−04
GA-11	0.06151041	1.23009392	−0.32203695	−0.84478930	0.03567868	2.5537E−04
GA-12	0.05962671	1.14482903	0.33821936	−0.83854375	0.03516478	3.9369E−04
GA-SQP-1	0.00275613	39.25753931	−0.06137573	0.85972527	0.03698507	6.6525E−16
GA-SQP-2	0.00311281	34.61266395	0.06502805	0.85937893	0.03695189	1.4845E−15
GA-SQP-3	0.00275605	39.25869183	−0.06137484	0.85972528	0.03698507	4.2918E−16
GA-SQP-4	0.00275616	39.25721209	−0.06137599	0.85972527	0.03698506	4.0331E−17
GA-SQP-5	0.00275615	39.25724045	−0.06137596	0.85972527	0.03698506	3.6564E−16
GA-SQP-6	0.00275614	39.25739493	−0.06137584	0.85972527	0.03698507	4.1288E−16
GA-SQP-7	0.00311297	34.61091320	0.06502969	0.85937892	0.03695189	2.6794E−16
GA-SQP-8	0.00311298	34.61075588	0.06502984	0.85937892	0.03695189	1.5478E−16
GA-SQP-9	0.00311294	34.61123963	0.06502938	0.85937892	0.03695189	1.3468E−15
GA-SQP-10	0.00275615	39.25730254	−0.06137592	0.85972527	0.03698506	4.0902E−16
GA-SQP-11	0.00275613	39.25760857	−0.06137568	0.85972527	0.03698507	6.9394E−16
GA-SQP-12	0.00311298	34.61075277	0.06502984	0.85937892	0.03695189	6.8128E−16

**Table 6:** Comparison of the performance on the basis of absolute values of constitutional equations of problem 3

Method	Absolute values					Method	Absolute values				
	$f_{1} ({\mathbf{t}})$	$f_{2} ({\mathbf{t}})$	$f_{3} ({\mathbf{t}})$	$f_{4} ({\mathbf{t}})$	$f_{5} ({\mathbf{t}})$		$f_{1} ({\mathbf{t}})$	$f_{2} ({\mathbf{t}})$	$f_{3} ({\mathbf{t}})$	$f_{4} ({\mathbf{t}})$	$f_{5} ({\mathbf{t}})$
GA-1	1.5E−02	8.7E−03	4.3E−03	4.1E−04	5.2E−04	GA-SQP-1	4.3E−08	9.2E−09	3.2E−08	3.5E−10	2.0E−08
GA-2	1.9E−02	9.9E−03	2.6E−03	2.9E−04	1.5E−03	GA-SQP-2	2.2E−07	1.2E−07	5.6E−08	3.1E−10	2.7E−09
GA-3	3.1E−02	1.7E−02	9.5E−03	1.9E−04	5.3E−04	GA-SQP-3	1.4E−07	8.2E−08	4.5E−08	2.7E−10	9.7E−10
GA-4	2.6E−02	1.6E−02	6.7E−03	1.6E−04	1.9E−04	GA-SQP-4	7.0E−09	1.0E−09	1.2E−08	9.2E−10	2.9E−10
GA-5	1.3E−02	7.2E−03	3.7E−03	2.8E−05	5.9E−05	GA-SQP-5	1.2E−08	2.4E−08	2.6E−08	1.8E−10	2.0E−08
GA-6	1.2E−02	4.5E−03	2.5E−03	5.9E−04	5.3E−03	GA-SQP-6	2.8E−08	1.5E−08	2.7E−08	3.4E−10	1.8E−08
GA-7	3.1E−02	1.6E−02	1.1E−02	5.5E−04	3.0E−03	GA-SQP-7	3.1E−08	1.3E−08	1.3E−08	3.2E−10	3.1E−09
GA-8	2.9E−02	2.1E−02	1.3E−02	1.7E−03	1.4E−03	GA-SQP-8	4.8E−09	2.6E−08	3.8E−09	1.7E−10	8.6E−09
GA-9	1.6E−02	9.4E−03	4.9E−03	1.2E−06	6.0E−04	GA-SQP-9	7.1E−08	1.6E−08	3.5E−08	2.9E−10	1.4E−08
GA-10	1.0E−02	1.5E−02	1.5E−02	4.0E−03	6.9E−03	GA-SQP-10	2.4E−08	2.6E−08	2.0E−08	4.6E−10	2.1E−08
GA-11	3.0E−02	1.7E−02	9.4E−03	1.6E−04	2.0E−03	GA-SQP-11	4.8E−08	4.7E−10	3.0E−08	3.4E−10	1.6E−08
GA -12	2.2E−02	2.4E−02	2.5E−02	3.1E−04	1.6E−02	GA-SQP-12	2.5E−08	3.1E−08	3.3E−08	3.0E−10	2.7E−08

### Problem 4: neurophysiology applications

In this case, the performance of the design scheme is examined on Bioinformatics problem based on neurophysiology applications (NPAs). Following a system of nonlinear equations representing NPA as (Grosan and Abraham 2008b; Verschelde et al. 1994):

10 $$\left\{ {\begin{array}{*{20}l} {f_{1} ({\mathbf{t}}) = t_{1}^{2} + t_{3}^{2} = 1} \hfill \\ {f_{2} ({\mathbf{t}}) = t_{2}^{2} + t_{4}^{2} = 1} \hfill \\ {f_{3} ({\mathbf{t}}) = t_{5} t_{3}^{2} + t_{6} t_{4}^{2} = c_{1} } \hfill \\ {f_{4} ({\mathbf{t}}) = t_{5} t_{1}^{3} + t_{6} t_{2}^{3} = c_{2} } \hfill \\ {f_{5} ({\mathbf{t}}) = t_{5} t_{1} t_{3}^{2} + t_{6} t_{4}^{2} t_{2} = c_{3} } \hfill \\ {f_{6} ({\mathbf{t}}) = t_{5} t_{1}^{2} t_{3} + t_{6} t_{2}^{2} t_{4} = c_{4} } \hfill \\ \end{array} } \right.,$$

where the values of constant c_1_ to c_4_ are chosen arbitrarily and ${\mathbf{t}} = \left( {t_{1} , \, t_{2} , \ldots ,t_{6} } \right)$. In model (10) the values of constant are taken zero. The design memetic computing approaches are applied to solve (10) by formulation of the fitness function in this case as:

11 $$\varepsilon = \frac{1}{6}\left( {\left( {f_{1} ({\mathbf{t}})} \right)^{2} + \left( {f_{2} ({\mathbf{t}})} \right)^{2} + \left( {f_{3} ({\mathbf{t}})} \right)^{2} + (f_{4} ({\mathbf{t}}))^{2} + \left( {f_{5} ({\mathbf{t}})} \right)^{2} + \left( {f_{6} ({\mathbf{t}})} \right)^{2} } \right).$$

Optimization of fitness function (11) is carried out with design variants of hybrid computing algorithms and results are tabulated in Table 7 along with the value of fitness and also in Table 8 for constitutional equations. It is seen that the values of fitness in case of GAs are around 10^−09^ to 10^−15^ while these values for the hybrid approach GA-SQP variants are around 10^−24^. Generally, observed that highly accurate results are determined by hybrid methodologies for this problem also.

**Table 7:** Comparison of trained parameters along with their fitness for GA and GA-SQP algorithms in case of problem 4

Method	Proposed solutions						*ɛ*
	*t*_1_	*t*_2_	*t*_3_	*t*_4_	*t*_5_	*t*_6_	
GA-1	0.46763361	−0.33451289	0.88392240	−0.94239114	−0.00000019	0.00000009	3.2086E−15
GA-2	0.99936492	−0.62752141	−0.03562736	0.77859916	−0.00000002	0.00000126	2.2147E−13
GA-3	0.24762472	0.95525282	0.96885624	−0.29579072	−0.00000035	0.00000019	5.1515E−14
GA-4	−0.05955973	−0.91317817	−0.99822579	0.40756671	0.00000306	−0.00000217	6.6386E−12
GA-5	−0.91497150	−0.59493980	−0.40351842	−0.80377028	0.00000008	−0.00000018	2.9425E−15
GA-6	0.00637146	0.92806866	0.99997967	−0.37240907	0.00000066	0.00000061	1.4258E−13
GA-7	0.70302906	−0.48804791	−0.71116111	0.87281683	0.00000002	−0.00000003	1.9908E−16
GA-8	−0.39538307	0.98389132	0.91850661	−0.17866286	0.00004137	−0.00000002	5.2853E−10
GA-9	−0.88160354	0.49496188	−0.47199049	−0.86891466	0.00000031	−0.00000009	1.7079E−14
GA-10	0.60385584	−0.97776199	0.79709341	−0.20971714	0.00000222	0.00000171	7.0492E−13
GA-11	−0.81473843	0.27084100	0.57982768	0.96262427	−0.00000213	0.00000511	3.8003E−12
GA-12	0.70613629	−0.93203731	−0.70808850	−0.36240691	0.00008539	−0.00002124	1.1376E−09
GA-SQP-1	0.28971734	0.00668370	0.95711225	0.99997766	0.00000000	0.00000000	8.4176E−24
GA-SQP-2	0.64464707	−0.70847890	−0.76448032	0.70573200	0.00000000	0.00000000	1.4960E−23
GA-SQP-3	0.21239967	0.58108779	−0.97718288	0.81384088	0.00000000	0.00000000	5.3650E−24
GA-SQP-4	−0.99315627	0.58140420	0.11679307	−0.81361487	0.00000000	0.00000000	1.0099E−23
GA-SQP-5	0.91699104	0.89690900	0.39890780	0.44221515	0.00000000	0.00000000	1.8177E−22
GA-SQP-6	−0.22521963	0.57489425	0.97430802	0.81822772	0.00000000	0.00000000	4.3799E−23
GA-SQP-7	−0.97134152	0.04316905	−0.23768813	0.99906778	0.00000000	0.00000000	3.1433E−23
GA-SQP-8	−0.57465370	−0.99701922	−0.81839668	−0.07715361	0.00000000	0.00000000	5.2944E−23
GA-SQP-9	0.81550584	−0.34778348	0.57874884	0.93757488	0.00000000	0.00000000	3.1435E−23
GA-SQP-10	−0.97410591	0.98078519	−0.22609217	0.19509079	0.00000000	0.00000000	1.5465E−23
GA-SQP-11	−0.60502940	−0.52408103	−0.79620313	0.85166840	0.00000000	0.00000000	1.0865E−22
GA-SQP-12	0.96895420	−0.90489448	−0.24724029	−0.42563598	0.00000000	0.00000000	1.4094E−23

**Table 8:** Comparison of the performance on the basis of absolute values of constitutional equations of problem 4

Method	Absolute values					
	$f_{1} ({\mathbf{t}})$	$f_{2} ({\mathbf{t}})$	$f_{3} ({\mathbf{t}})$	$f_{4} ({\mathbf{t}})$	$f_{5} ({\mathbf{t}})$	$f_{6} ({\mathbf{t}})$
GA-1	2.99E−09	5.85E−08	6.21E−08	2.27E−08	9.66E−08	4.62E−08
GA-2	4.46E−07	2.28E−07	7.66E−07	3.32E−07	4.80E−07	3.88E−07
GA-3	4.12E−07	9.72E−08	3.08E−07	1.61E−07	6.44E−08	7.22E−08
GA-4	2.08E−06	5.00E−06	2.69E−06	1.65E−06	1.48E−07	7.48E−07
GA-5	4.00E−08	3.44E−08	1.02E−07	2.48E−08	5.67E−08	2.34E−08
GA-6	5.64E−08	5.66E−08	7.50E−07	4.91E−07	8.33E−08	1.97E−07
GA-7	2.10E−08	1.22E−08	1.24E−08	8.73E−09	1.57E−08	1.13E−08
GA-8	1.78E−05	3.74E−05	3.49E−05	2.57E−06	1.38E−05	5.94E−06
GA-9	1.77E−07	4.37E−08	7.95E−10	2.26E−07	9.64E−08	9.50E−08
GA-10	2.15E−07	2.14E−07	1.48E−06	1.11E−06	7.78E−07	3.02E−07
GA-11	1.14E−06	3.37E−07	4.02E−06	1.25E−06	1.86E−06	4.58E−07
GA-12	1.78E−05	3.23E−05	4.00E−05	4.73E−05	3.28E−05	2.35E−05
GA-SQP-1	5.72E−12	4.20E−12	3.08E−13	8.00E−15	8.73E−14	2.64E−14
GA-SQP-2	3.67E−11	3.63E−11	7.92E−13	9.68E−13	1.21E−12	1.08E−12
GA-SQP-3	1.17E−12	1.33E−11	7.56E−12	4.28E−12	7.02E−12	6.43E−12
GA-SQP-4	7.27E−12	1.91E−12	1.64E−12	2.48E−13	9.48E−13	6.52E−13
GA-SQP-5	4.56E−13	3.24E−11	2.31E−12	4.38E−12	2.00E−12	3.16E−12
GA-SQP-6	6.32E−13	1.44E−11	6.68E−12	1.32E−12	2.29E−12	2.02E−12
GA-SQP-7	5.52E−12	8.59E−12	1.05E−13	8.91E−12	5.52E−13	2.18E−12
GA-SQP-8	4.19E−12	1.64E−11	4.45E−12	1.27E−12	2.56E−12	1.80E−12
GA-SQP-9	4.80E−12	1.25E−11	2.00E−12	1.55E−12	1.71E−12	6.34E−13
GA-SQP-10	8.82E−12	3.30E−12	1.07E−12	1.58E−12	2.31E−13	7.05E−13
GA-SQP-11	6.73E−12	1.04E−11	1.22E−11	6.17E−12	8.42E−12	1.55E−11
GA-SQP-12	6.51E−12	3.04E−13	2.06E−12	3.33E−12	7.03E−13	5.12E−12

### Problem 5: combustion theory applications

Well-known problem of combustion theory with temperature around 3000 °C is taken in this case (Morgan 1987; Grosan and Abraham 2008b). Governing mathematical relations for the problem in term of system of nonlinear equations is given as follows:

12 $$\left\{ {\begin{array}{*{20}l} {f_{1} ({\mathbf{t}}) = t_{2} + 2t_{6} + t_{9} + 2t_{10} = 10^{ - 5} } \hfill \\ {f_{2} ({\mathbf{t}}) = t_{3} + t_{8} = 3 \times 10^{ - 5} } \hfill \\ {f_{3} ({\mathbf{t}}) = t_{1} + t_{3} + 2t_{5} + 2t_{8} + t_{9} + t_{10} = 5 \times 10^{ - 5} } \hfill \\ {f_{4} ({\mathbf{t}}) = t_{4} + 2t_{7} = 10^{ - 5} } \hfill \\ {f_{5} ({\mathbf{t}}) = 0.5140437\, \times 10^{ - 7} t_{5} = t_{1}^{2} } \hfill \\ {f_{6} ({\mathbf{t}}) = 0.1006932 \times 10^{ - 6} t_{6} = 2t_{2}^{2} } \hfill \\ {f_{7} ({\mathbf{t}}) = 0.7816278 \times 10^{ - 15} t_{7} = t_{4}^{2} } \hfill \\ {f_{8} ({\mathbf{t}}) = 0.1496236 \times 10^{ - 6} t_{8} = t_{1} t_{3} } \hfill \\ {f_{9} ({\mathbf{t}}) = 0.6194411 \times 10^{ - 7} t_{9} = t_{1} t_{2} } \hfill \\ {f_{10} ({\mathbf{t}}) = 0.2089296 \times 10^{ - 14} t_{10} = t_{1} t_{2}^{2} } \hfill \\ \end{array} } \right.$$

Here, the input vector ${\mathbf{t}} = \left( {t_{1} , \, t_{2} , \ldots ,t_{10} } \right)$. The design computing approaches are applied to this case by constructing a fitness function as given below:

13 $$\varepsilon = \frac{1}{10}\left( {\left( {f_{1} ({\mathbf{t}})} \right)^{2} + \left( {f_{2} ({\mathbf{t}})} \right)^{2} + \left( {f_{3} ({\mathbf{t}})} \right)^{2} + , \ldots , + \left( {f_{10} ({\mathbf{t}})} \right)^{2} } \right).$$

**Fig. 4:**
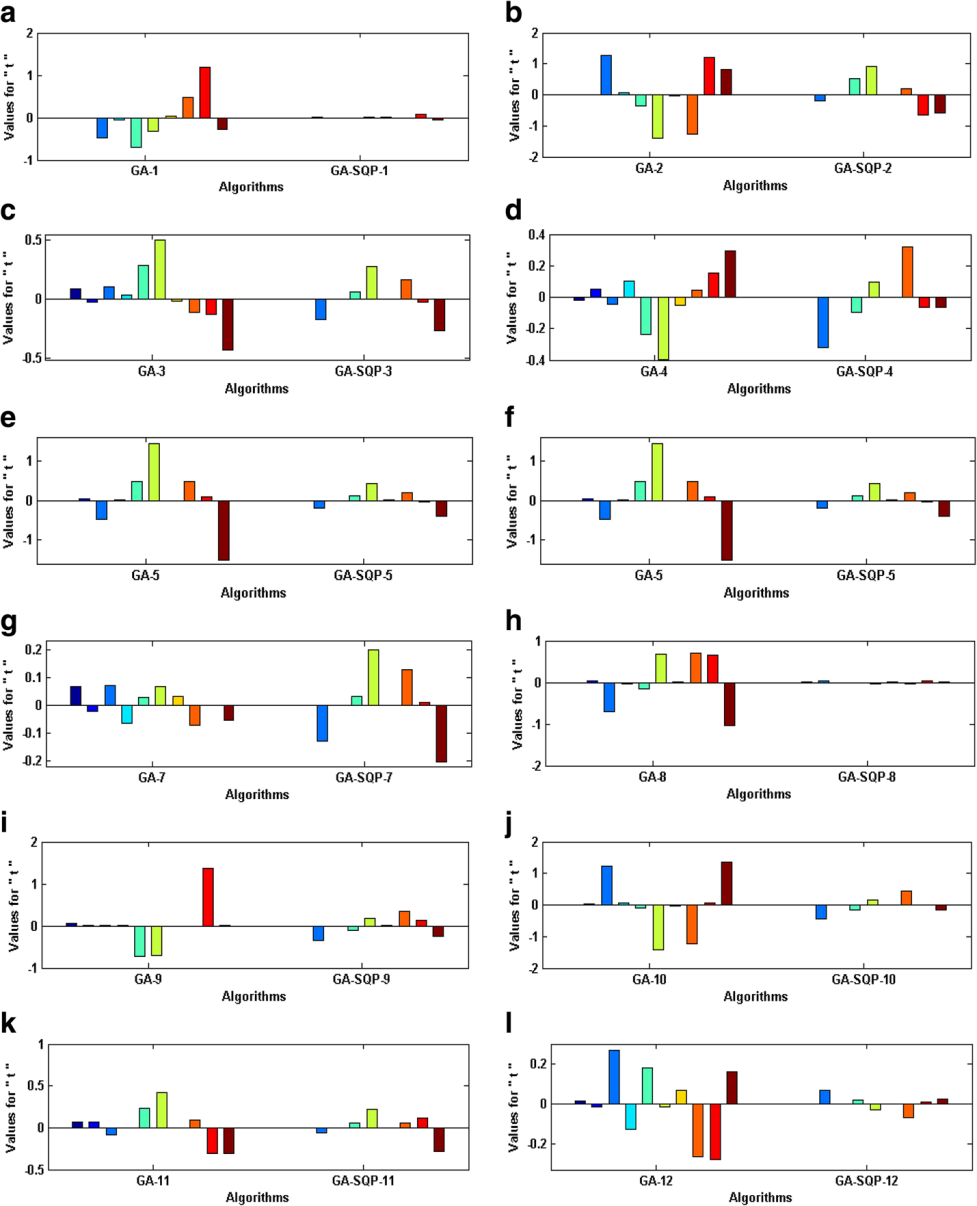
Trained parameters of GA and GA-SQP algorithms in case of problem *5*, while the figures (**a**–**l**), represent the results of variant *1*–*12* of proposed methodology, respectively

Optimization of fitness function (13) is carried out with the designed schemes and results are represented graphically in Fig. 4 for variants of GAs and GA-SQP algorithms. The absolute values of constitutional equations are calculated and results are tabulated in Table 9 for each variant. It is seen that the maximum values of $f_{i} ({\mathbf{t}})$, *i* = 1–10, for GA and GA-SQP algorithms are 1.00 × 10^−09^ and 1.15 × 10^−08^, respectively. It is observed that generally hybrid computing approaches outperformed the rest of techniques.

**Table 9:** Comparison of the performance on the basis of absolute values of constitutional equations of problem 5

Method	Absolute values									
	$f_{1} ({\mathbf{t}})$	$f_{2} ({\mathbf{t}})$	$f_{3} ({\mathbf{t}})$	$f_{4} ({\mathbf{t}})$	$f_{5} ({\mathbf{t}})$	$f_{6} ({\mathbf{t}})$	$f_{7} ({\mathbf{t}})$	$f_{8} ({\mathbf{t}})$	$f_{9} ({\mathbf{t}})$	$f_{10} ({\mathbf{t}})$
GA-1	2.34E−12	4.11E−12	1.33E−12	4.96E−13	3.16E−09	1.34E−09	4.15E−09	8.55E−12	1.38E−09	1.63E−15
GA-2	2.22E−13	2.55E−12	5.55E−13	1.33E−13	4.83E−09	5.90E−10	1.98E−09	2.14E−12	3.85E−09	6.43E−16
GA-3	3.21E−12	4.13E−12	3.70E−12	1.50E−12	6.53E−09	1.78E−10	9.56E−10	4.33E−12	1.93E−09	2.33E−15
GA-4	3.40E−12	1.54E−11	3.21E−12	1.53E−12	3.60E−09	4.19E−10	2.46E−09	2.81E−11	1.15E−08	2.98E−15
GA-5	1.43E−12	2.36E−13	6.44E−12	1.58E−12	1.67E−09	1.46E−10	1.00E−09	3.36E−12	5.68E−10	1.07E−15
GA-6	9.49E−14	1.24E−13	6.97E−14	9.58E−15	4.29E−10	3.29E−09	6.22E−10	4.71E−13	2.49E−09	2.95E−17
GA-7	2.63E−12	2.38E−12	3.20E−12	2.40E−12	6.10E−09	8.68E−10	1.75E−09	5.29E−13	8.11E−09	8.00E−16
GA-8	2.18E−12	3.48E−12	7.63E−12	2.96E−12	7.82E−09	3.49E−10	1.38E−09	1.43E−11	3.93E−10	8.13E−16
GA-9	8.43E−13	2.10E−12	9.20E−13	7.86E−12	2.71E−09	2.04E−11	2.43E−10	7.98E−12	7.34E−09	1.09E−15
GA-10	3.26E−12	5.36E−12	7.68E−12	4.39E−13	8.81E−10	3.05E−09	1.23E−09	1.81E−11	5.57E−10	5.88E−17
GA-11	2.34E−12	4.11E−12	1.33E−12	4.96E−13	3.16E−09	1.34E−09	4.15E−09	8.55E−12	1.38E−09	1.63E−15
GA-12	2.22E−13	2.55E−12	5.55E−13	1.33E−13	4.83E−09	5.90E−10	1.98E−09	2.14E−12	3.85E−09	6.43E−16
GA-SQP-1	3.21E−12	4.13E−12	3.70E−12	1.50E−12	6.53E−09	1.78E−10	9.56E−10	4.33E−12	1.93E−09	2.33E−15
GA-SQP-2	3.40E−12	1.54E−11	3.21E−12	1.53E−12	3.60E−09	4.19E−10	2.46E−09	2.81E−11	1.15E−08	2.98E−15
GA-SQP-3	1.43E−12	2.36E−13	6.44E−12	1.58E−12	1.67E−09	1.46E−10	1.00E−09	3.36E−12	5.68E−10	1.07E−15
GA-SQP-4	9.49E−14	1.24E−13	6.97E−14	9.58E−15	4.29E−10	3.29E−09	6.22E−10	4.71E−13	2.49E−09	2.95E−17
GA-SQP-5	2.63E−12	2.38E−12	3.20E−12	2.40E−12	6.10E−09	8.68E−10	1.75E−09	5.29E−13	8.11E−09	8.00E−16
GA-SQP-6	2.18E−12	3.48E−12	7.63E−12	2.96E−12	7.82E−09	3.49E−10	1.38E−09	1.43E−11	3.93E−10	8.13E−16
GA-SQP-7	8.43E−13	2.10E−12	9.20E−13	7.86E−12	2.71E−09	2.04E−11	2.43E−10	7.98E−12	7.34E−09	1.09E−15
GA-SQP-8	3.26E−12	5.36E−12	7.68E−12	4.39E−13	8.81E−10	3.05E−09	1.23E−09	1.81E−11	5.57E−10	5.88E−17
GA-SQP-9	2.34E−12	4.11E−12	1.33E−12	4.96E−13	3.16E−09	1.34E−09	4.15E−09	8.55E−12	1.38E−09	1.63E−15
GA-SQP-10	2.22E−13	2.55E−12	5.55E−13	1.33E−13	4.83E−09	5.90E−10	1.98E−09	2.14E−12	3.85E−09	6.43E−16
GA-SQP-11	3.21E−12	4.13E−12	3.70E−12	1.50E−12	6.53E−09	1.78E−10	9.56E−10	4.33E−12	1.93E−09	2.33E−15
GA-SQP-12	3.40E−12	1.54E−11	3.21E−12	1.53E−12	3.60E−09	4.19E−10	2.46E−09	2.81E−11	1.15E−08	2.98E−15

### Problem 6: economics modelling application

Another problem is selected for the study arising in econometric modelling applications (EMAs) extensively based on system of nonlinear equation of arbitrary dimensions (Morgan 1987; Grosan and Abraham 2008b). These problems are generally considered to be stiff to solve numerically and given in terms of mathematical relations as:

14 $$\left\{ {\begin{array}{*{20}l} {\left( {t_{k} + \sum\limits_{i = 1}^{n - k - 1} {t_{i} t_{i} + k} } \right)t_{n} - c_{k} = 0;\quad 1 \le k \le n - 1} \hfill \\ {\sum\limits_{i = 1}^{n - 1} {t_{i} + 1} = 0.} \hfill \\ \end{array} } \right.,$$

here the value of “c_k_” can be randomly chosen. A special case of the problem (14) is taken using *n* = 5 and *k* = 1 to 4 for evaluation of the proposed algorithms and its governing nonlinear system of the equations are written as:

15 $$\left\{ {\begin{array}{*{20}l} {f_{1} ({\mathbf{t}}) = t_{1} + t_{2} + t_{3} + t_{4} = - 1} \hfill \\ {f_{2} ({\mathbf{t}}) = (t_{1} + t_{1} t_{2} + t_{2} t_{3} + t_{3} t_{4} )t_{5} = 1\quad\,for\,k = 1} \hfill \\ {f_{3} ({\mathbf{t}}) = (t_{2} + t_{1} t_{3} + t_{2} t_{4} )t_{5} = 1\quad\,for\,k = 2} \hfill \\ {f_{4} ({\mathbf{t}}) = (t_{3} + t_{1} t_{4} )t_{5} \, = \,1\quad\,for\,k\, = \,3} \hfill \\ {f_{5} ({\mathbf{t}}) = t_{4} t_{5} = 1\quad\,for\,k\, = 4} \hfill \\ \end{array} } \right.,$$

where ${\mathbf{t}} = \left( {t_{1} , \, t_{2} , \ldots ,t_{5} } \right)$. The design approaches are applied to solve the system (15) with the help of optimization of the fitness function as defined below:

16 $$\varepsilon = \frac{1}{5}\left( {\left( {f_{1} ({\mathbf{t}})} \right)^{2} + \left( {f_{2} ({\mathbf{t}})} \right)^{2} + \left( {f_{3} ({\mathbf{t}})} \right)^{2} \left( {f_{4} ({\mathbf{t}})} \right)^{2} + \left( {f_{5} ({\mathbf{t}})} \right)^{2} } \right).$$

Results of proposed adaptive algorithms are tabulated in Tables 10 and 11 and these results show that values of fitness *ε* for GAs lie around 10^−07^ to 10^−10^ while these values for GA-SQP variants are around 10^−33^. Generally, it is observed that the most accurate results are determined by hybrid computing platforms.

**Table 10:** Comparison of trained parameters along with their fitness for GA and GA-SQP algorithms in case of problem 6

Method	Proposed solutions					*ɛ*
	*t*_1_	*t*_2_	*t*_3_	*t*_4_	*t*_5_	
GA-1	−0.05232134	2.55174864	−1.79439654	−1.70502885	−0.58646497	1.2304E−09
GA-2	−0.05235315	2.55165541	−1.79424191	−1.70503153	−0.58650707	4.9779E−10
GA-3	−0.05237792	2.54633426	−1.79043794	−1.70193969	−0.58882572	3.6107E−06
GA-4	1.04961877	−1.49514638	0.03145951	−0.58636170	−1.70890458	1.7101E−06
GA-5	1.05237831	−1.49657666	0.03069550	−0.58650558	−1.70496102	2.3976E−10
GA-6	−0.05239351	2.55146647	−1.79407554	−1.70496963	−0.58658463	3.0835E−09
GA-7	1.05155963	−1.49559735	0.03101468	−0.58635525	−1.70711513	3.3197E−07
GA-8	1.04986636	−1.49478395	0.03130816	−0.58633776	−1.70956928	1.6279E−06
GA-9	−0.34925861	−0.27768182	−0.21422258	−0.15877634	−6.29819537	1.1820E−09
GA-10	−0.05251523	2.55075517	−1.79319677	−1.70475423	−0.58687119	9.4592E−08
GA-11	1.05132700	−1.49484237	0.03124121	−0.58622944	−1.70773132	9.0707E−07
GA-12	1.05401533	−1.49771615	0.03017421	−0.58658770	−1.70156701	8.8953E−07
GA-SQP-1	−0.34928445	−0.27770053	−0.21423688	−0.15877814	−6.29809606	4.9304E−33
GA-SQP-2	−0.34928445	−0.27770053	−0.21423688	−0.15877814	−6.29809606	9.8608E−33
GA-SQP-3	−0.05235137	2.55164291	−1.79427573	−1.70501581	−0.58650482	2.4652E−33
GA-SQP-4	−0.34928445	−0.27770053	−0.21423688	−0.15877814	−6.29809606	9.8608E−33
GA-SQP-5	1.34928445	1.74898460	2.19982701	−6.29809606	−0.15877814	9.8608E−33
GA-SQP-6	−0.05235137	2.55164291	−1.79427573	−1.70501581	−0.58650482	9.8608E−33
GA-SQP-7	−0.05235137	2.55164291	−1.79427573	−1.70501581	−0.58650482	9.8608E−33
GA-SQP-8	−0.34928445	−0.27770053	−0.21423688	−0.15877814	−6.29809606	2.4652E−33
GA-SQP-9	1.34928445	1.74898460	2.19982701	−6.29809606	−0.15877814	4.9304E−33
GA-SQP-10	−0.05235137	2.55164291	−1.79427573	−1.70501581	−0.58650482	9.8608E−33
GA-SQP-11	−0.34928445	−0.27770053	−0.21423688	−0.15877814	−6.29809606	2.4652E−33
GA-SQP-12	−0.34928445	−0.27770053	−0.21423688	−0.15877814	−6.29809606	2.4652E−33

**Table 11:** Comparison of the performance on the basis of absolute values of constitutional equations of problem 6

Method	Absolute values					Method	Absolute values				
	$f_{1} ({\mathbf{t}})$	$f_{2} ({\mathbf{t}})$	$f_{3} ({\mathbf{t}})$	$f_{4} ({\mathbf{t}})$	$f_{5} ({\mathbf{t}})$		$f_{1} ({\mathbf{t}})$	$f_{2} ({\mathbf{t}})$	$f_{3} ({\mathbf{t}})$	$f_{4} ({\mathbf{t}})$	$f_{5} ({\mathbf{t}})$
GA-1	1.9E−06	3.0E−05	2.3E−05	3.3E−05	6.0E−05	GA-SQP-1	1.1E−16	0.0E+00	0.0E+00	0.0E+00	1.1E−16
GA-2	2.9E−05	1.2E−05	3.2E−05	1.8E−05	1.3E−05	GA-SQP-2	2.2E−16	0.0E+00	0.0E+00	0.0E+00	1.1E−16
GA-3	1.6E−03	4.2E−04	2.8E−03	1.8E−03	2.1E−03	GA-SQP-3	0.0E+00	2.2E−16	4.4E−16	2.2E−16	0.0E+00
GA-4	4.3E−04	4.8E−05	4.4E−04	2.0E−03	2.0E−03	GA-SQP-4	0.0E+00	0.0E+00	0.0E+00	2.2E−16	0.0E+00
GA-5	8.4E−06	7.0E−06	1.5E−06	1.1E−05	3.1E−05	GA-SQP-5	0.0E+00	2.2E−16	0.0E+00	0.0E+00	0.0E+00
GA-6	2.8E−05	1.8E−05	4.4E−05	2.2E−05	1.1E−04	GA-SQP-6	0.0E+00	2.2E−16	0.0E+00	0.0E+00	0.0E+00
GA-7	6.2E−04	1.1E−04	4.2E−04	3.6E−04	9.8E−04	GA-SQP-7	0.0E+00	2.2E−16	0.0E+00	0.0E+00	0.0E+00
GA-8	5.3E−05	5.6E−04	9.0E−04	1.2E−03	2.4E−03	GA-SQP-8	1.1E−16	0.0E+00	0.0E+00	0.0E+00	0.0E+00
GA-9	6.1E−05	6.4E−06	1.4E−05	4.4E−05	4.4E−06	GA-SQP-9	0.0E+00	0.0E+00	0.0E+00	1.1E−16	1.1E−16
GA-10	2.9E−04	2.6E−04	2.7E−04	1.6E−04	4.7E−04	GA-SQP-10	2.2E−16	0.0E+00	0.0E+00	0.0E+00	0.0E+00
GA-11	1.5E−03	5.4E−04	1.8E−04	8.4E−04	1.1E−03	GA-SQP-11	0.0E+00	0.0E+00	0.0E+00	1.1E−16	0.0E+00
GA -12	1.1E−04	3.4E−04	5.5E−04	6.9E−04	1.9E−03	GA-SQP-12	0.0E+00	0.0E+00	0.0E+00	0.0E+00	1.1E−16

## Comparative studies

In this section, comparative studies based on the results of statistical analysis are presented for variants of GA and GA-SQP for all the six systems of nonlinear equations. These analyses are used to draw reliable and constructive inferences on the performance of designed algorithms.

### Statistical performance indicators

Statistical performance indicator base on mean and standard deviation (STD) are used to analyze the performance for each variant of hybrid technique GA-SQP for 100 independent runs of the algorithm to solve all six nonlinear equations. Results based on the data generated from these simulations are used to draw a constructive and effective inference for the performance of each algorithm.

Hundred independent runs for all 12 proposed schemes are performed to determine the solution of six problems based on nonlinear equations using the similar procedure as adopted in the last section. Results based on values of statistical indices are given in Table 12, while the values based on truncated data for 75 best runs, i.e., the run with minimum fitness values. Analysis based on truncated runs is given due to the fact that one single bad run of the algorithm spoiled all the results. It is observed that the values of mean for GA, GA-SQP variants lie in the range of 1.1 × 10^−02^ to 4.4 × 10^−07^ and 1.2 × 10^−02^ to 1.9 × 10^−32^ respectively, while from Table 12 these respective values lie in the range of 1.0 × 10^−03^ to 1.1 × 10^−09^ and 1.2 × 10^−08^ to 9.1 × 10^−32^. Small values of the mean along with its STD are generally observed from each hybrid computing mechanism.

**Table 12:** Comparison of results on the basis of statistical performance indices for truncated 75% independent runs with better fitness

Methods	Problem-1		Problem-2		Problem-3		Problem-4		Problem-5		Problem-6	
	Mean	STD	Mean	STD	Mean	STD	Mean	STD	Mean	STD	Mean	STD
GA-1	3.6E−03	1.6E−03	1.5E−07	9.4E−08	8.5E−04	3.6E−04	5.5E−10	1.2E−09	5.3E−05	3.2E−05	5.6E−05	1.5E−04
GA-2	2.6E−03	1.7E−03	1.9E−07	1.2E−07	5.5E−03	6.1E−03	2.2E−08	7.5E−08	2.8E−05	1.6E−05	2.4E−05	4.0E−05
GA-3	5.4E−03	1.3E−03	7.3E−04	6.1E−04	1.9E−03	1.0E−03	5.7E−07	1.6E−06	1.0E−03	9.0E−04	2.0E−03	3.4E−03
GA-4	5.2E−03	1.7E−03	5.3E−06	3.3E−06	5.5E−03	5.3E−03	3.3E−07	5.6E−07	1.2E−04	6.7E−05	8.6E−04	8.1E−04
GA-5	3.3E−03	1.7E−03	1.2E−07	8.1E−08	8.2E−04	3.9E−04	9.3E−11	2.4E−10	5.4E−05	2.8E−05	4.1E−05	1.2E−04
GA-6	2.8E−03	1.8E−03	1.6E−07	9.6E−08	5.6E−03	6.4E−03	1.6E−09	6.2E−09	3.0E−05	1.6E−05	1.6E−05	2.4E−05
GA-7	5.4E−03	1.5E−03	5.0E−04	4.6E−04	1.7E−03	7.7E−04	3.1E−07	5.8E−07	6.5E−04	5.1E−04	9.5E−04	1.8E−03
GA-8	5.2E−03	1.6E−03	4.9E−06	3.1E−06	7.4E−03	6.8E−03	1.5E−06	3.0E−06	1.1E−04	6.0E−05	1.1E−03	1.2E−03
GA-9	3.5E−03	1.9E−03	7.0E−07	4.1E−07	1.0E−03	4.7E−04	1.1E−09	2.4E−09	8.1E−05	4.5E−05	5.5E−05	8.3E−05
GA-10	3.1E−03	1.9E−03	1.1E−06	5.5E−07	5.7E−03	7.2E−03	1.3E−07	3.8E−07	5.4E−05	3.1E−05	3.6E−04	1.0E−03
GA-11	5.6E−03	1.2E−03	1.4E−03	9.7E−04	2.1E−03	1.0E−03	8.1E−07	1.5E−06	1.1E−03	9.5E−04	5.9E−03	1.4E−02
GA-12	5.3E−03	1.7E−03	2.9E−05	1.8E−05	7.5E−03	6.5E−03	1.2E−06	2.0E−06	2.1E−04	1.3E−04	2.7E−03	4.1E−03
GA-SQP-1	6.9E−17	1.7E−17	1.8E−32	7.0E−33	3.1E−08	5.7E−08	3.9E−22	1.0E−22	1.2E−15	1.3E−15	9.7E−32	6.2E−32
GA-SQP-2	7.0E−17	1.8E−17	1.8E−32	6.8E−33	4.1E−08	6.7E−08	4.9E−22	2.9E−22	7.4E−16	5.9E−16	9.5E−32	6.6E−32
GA-SQP-3	7.0E−17	1.7E−17	1.6E−32	5.8E−33	6.2E−09	1.2E−08	4.0E−22	8.8E−23	2.4E−16	3.2E−16	1.1E−31	8.9E−32
GA-SQP-4	6.5E−17	1.8E−17	1.6E−32	7.2E−33	8.4E−09	1.3E−08	3.4E−22	1.3E−22	2.0E−16	2.5E−16	1.0E−31	6.5E−32
GA-SQP-5	6.6E−17	1.5E−17	2.0E−32	8.6E−33	4.1E−08	7.0E−08	4.0E−22	6.8E−23	6.7E−16	6.6E−16	1.1E−31	7.5E−32
GA-SQP-6	6.9E−17	1.7E−17	1.7E−32	8.0E−33	4.2E−08	7.0E−08	8.0E−22	1.3E−21	1.0E−15	1.1E−15	9.2E−32	5.8E−32
GA-SQP-7	6.7E−17	1.8E−17	1.4E−32	5.4E−33	1.2E−08	3.0E−08	4.0E−22	8.2E−23	2.5E−16	3.6E−16	8.0E−32	5.8E−32
GA-SQP-8	6.9E−17	1.8E−17	1.6E−32	6.0E−33	3.5E−08	6.4E−08	3.6E−22	1.2E−22	2.7E−16	4.3E−16	9.1E−32	6.6E−32
GA-SQP-9	6.9E−17	1.6E−17	1.7E−32	7.4E−33	3.4E−08	6.2E−08	3.9E−22	1.2E−22	8.4E−16	1.0E−15	7.8E−32	5.1E−32
GA-SQP-10	6.7E−17	1.7E−17	1.8E−32	7.5E−33	4.9E−08	7.2E−08	1.3E−21	2.2E−21	5.4E−16	4.4E−16	8.2E−32	5.7E−32
GA-SQP-11	6.8E−17	1.5E−17	1.4E−32	5.6E−33	8.8E−09	1.3E−08	3.9E−22	8.8E−23	4.7E−16	6.5E−16	8.5E−32	6.5E−32
GA-SQP-12	6.7E−17	1.9E−17	1.4E−32	6.0E−33	4.4E−08	6.9E−08	3.6E−22	1.4E−22	3.0E−16	4.2E−16	8.7E−32	7.1E−32

**Fig. 5:**
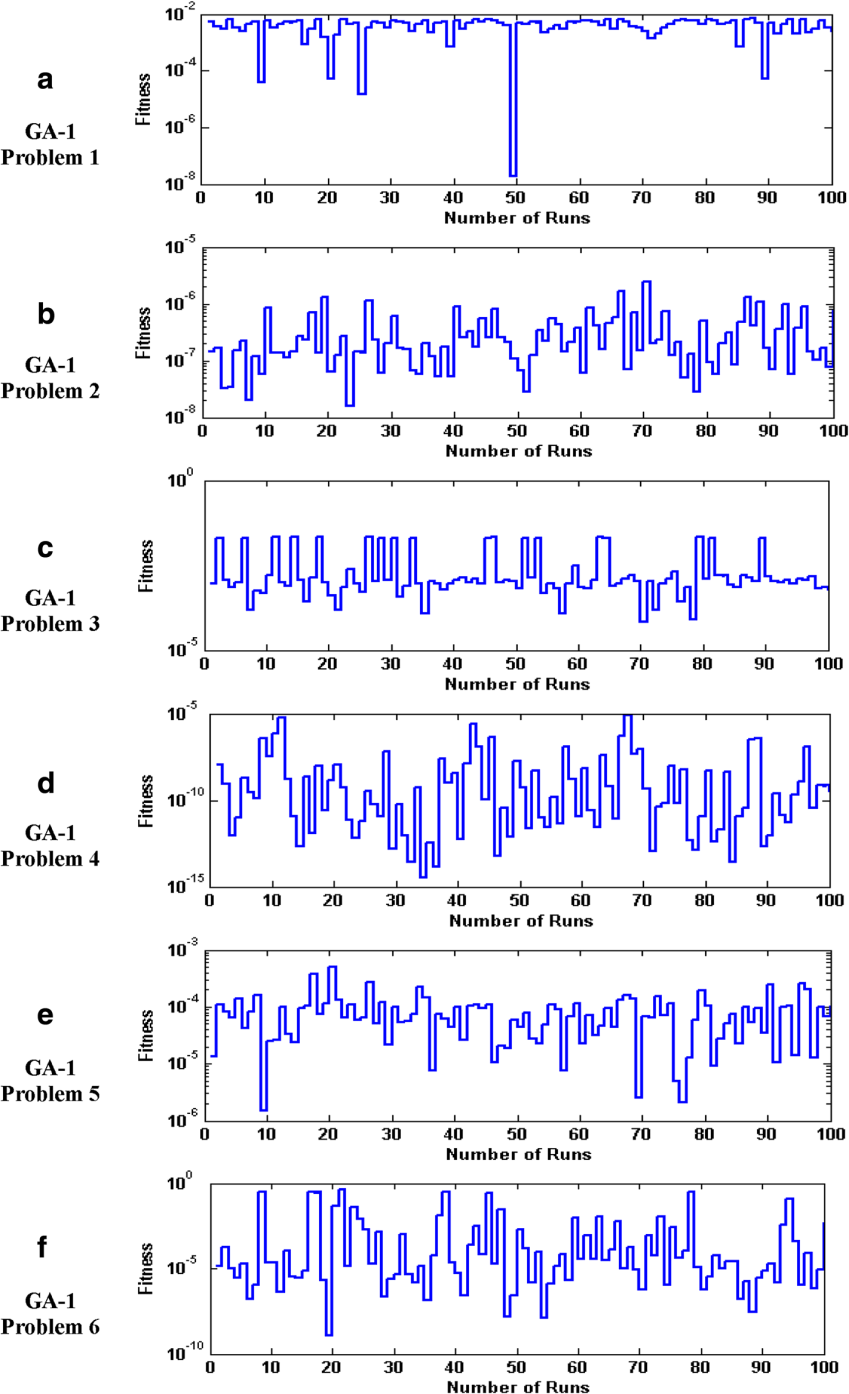
Plot of fitness against hundred independent runs for GA-1 algorithm in case of all six problems, while the figures (**a**–**f**) represent the results of problems *1*–*6*, respectively

**Fig. 6:**
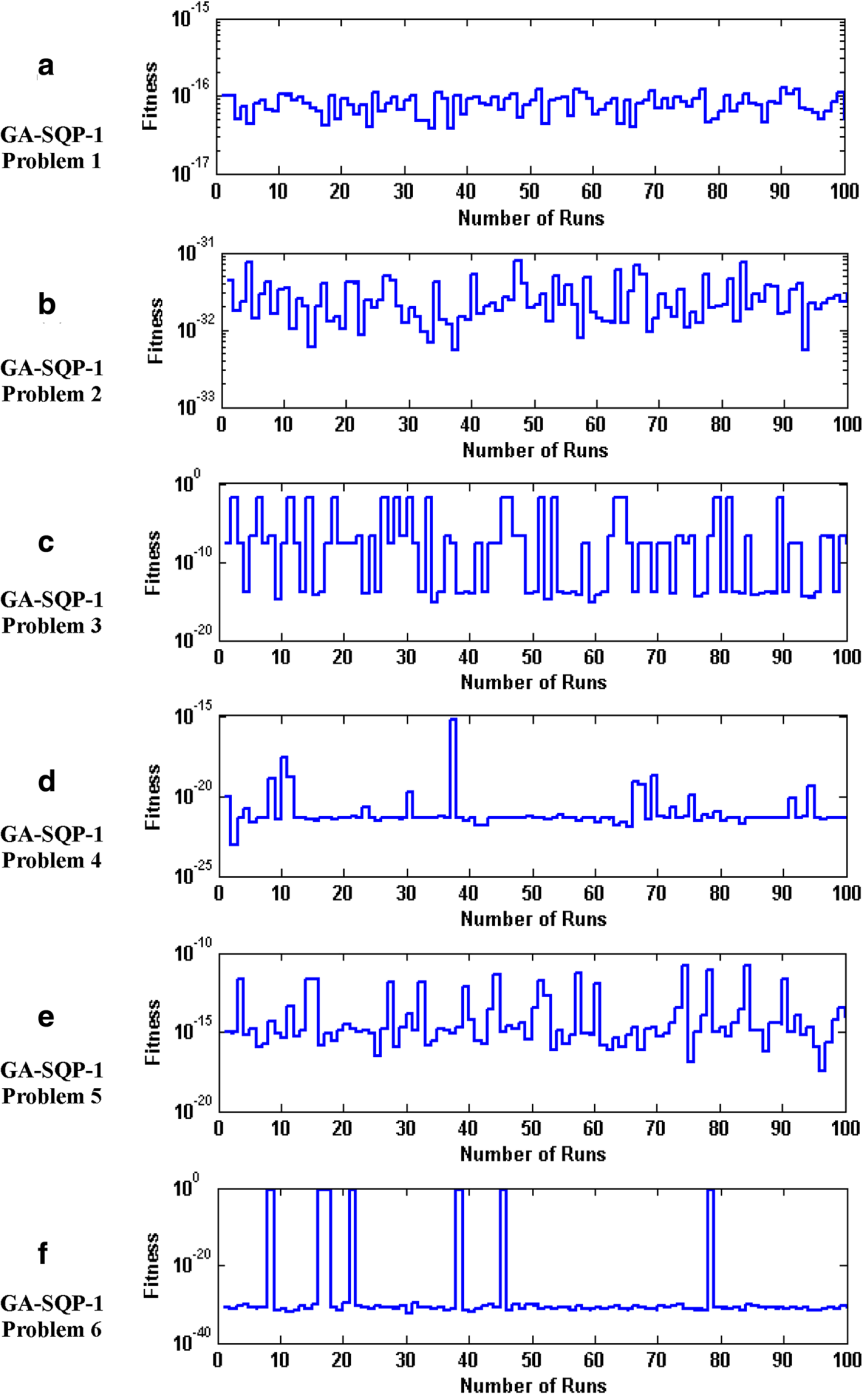
Plot of fitness against hundred independent runs for GA-SQP-1 algorithm in case of all six problems, while the figures (**a**–**f**) represent the results of problems *1*–*6*, respectively

**Fig. 7:**
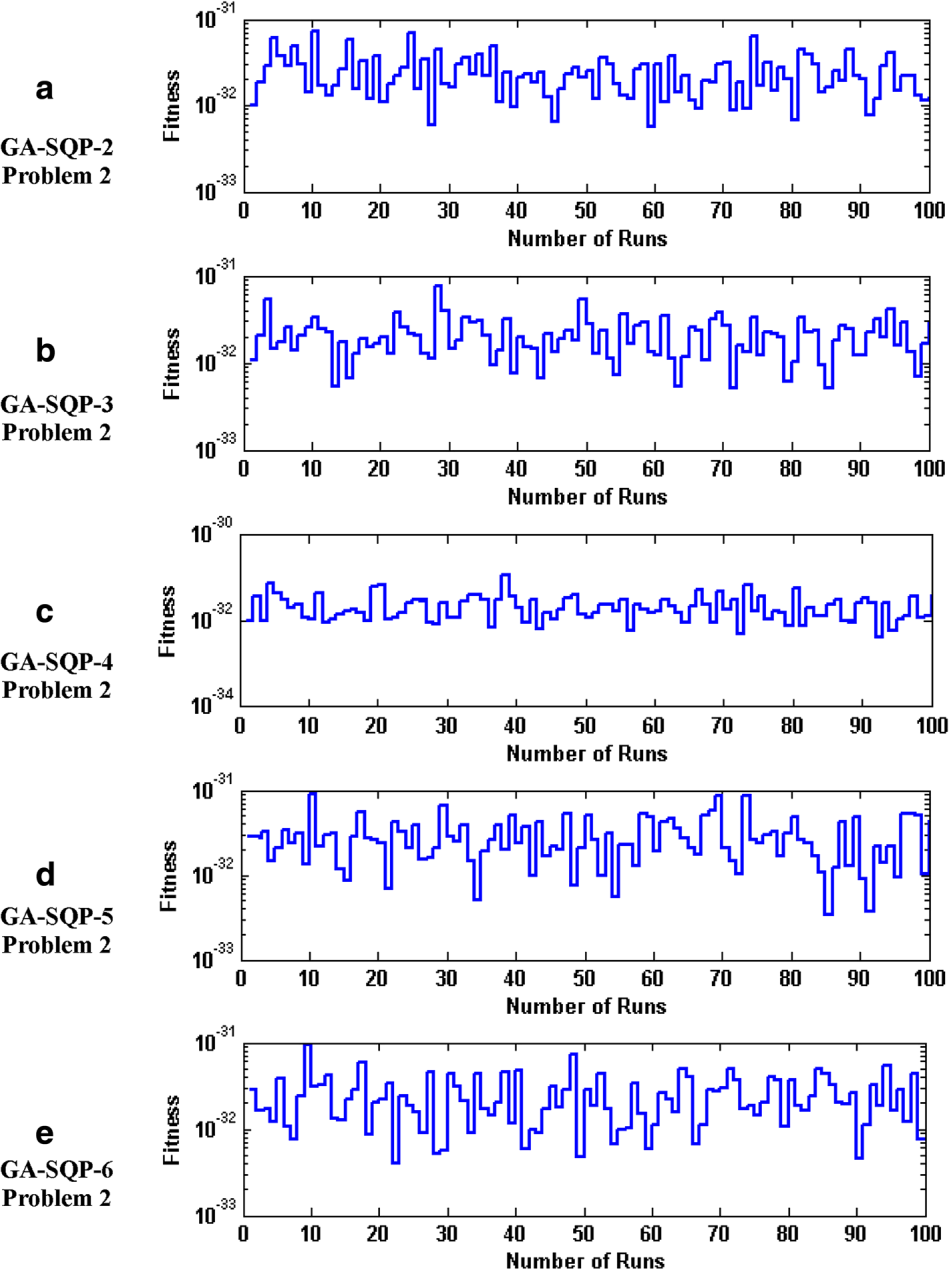
Plot of fitness against hundred independent runs for variants of GA-SQP algorithm in case of problem 2, while the figures (**a**–**e**), represent the results of variant *2*–*6*, respectively

**Fig. 8:**
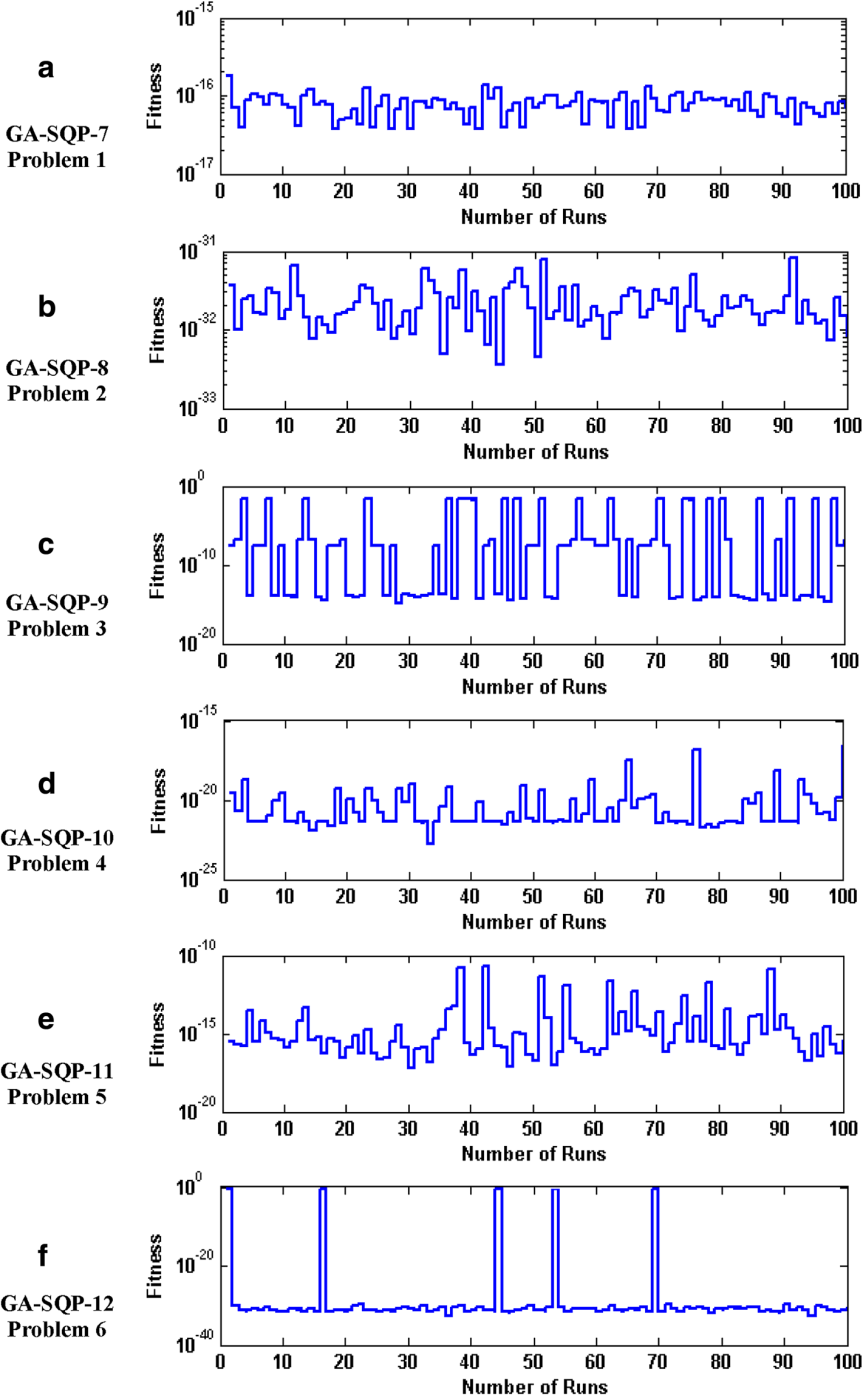
Plot of fitness against hundred independent runs for variants of GA-SQP algorithm in case of all six problems, while the figures (**a**–**f**), represent the results of variant of problem *1*–*6*, respectively

The accuracy and convergence of the proposed design hybrid schemes are analyzed based on 100 independent executions of algorithms. Results on the basis of value of fitness *ε* against number of independent runs on semi-logarithmic scale are plotted for GA-1 and GA-SQP-1 for all six problems in Figs. 5 and 6, respectively, while the results for the variants of hybrid computing approaches in case of problem 2 are plotted in Fig. 7. Moreover, graphical illustrations of results in cases of different algorithms on different problems are given in Fig. 8. It is seen that the performance of magnetic versions is much superior to a variant of GAs. For first five problems, almost 100% convergent and accurate solutions are found by the hybrid schemes, however, very few, i.e., less than 5% runs, are observed to trap in premature convergence in case of problem 6. The most accurate results are found for problem 2, 4 and 6 by the design hybrid computing techniques.

Results of proposed variants of GA-SQP are compared with simulated annealing (SA), pattern-search (PS), Nelder–Mead (NM) and Levenberg–Marquardt (LM) algorithm. Results of SA, PS, NM and LM methods are determined using built-in functions in Matlab environment for all six problems of nonlinear system of equations. The mean fitness values based on 20 runs of the SA, PS, NM and LM approaches lies in the range 10^−02^ to 10^−04^, 10^−02^ to 10^−05^, 10^−03^ to 10^−17^ and 10^−02^ to 10^−03^, respectively. It is clearly inferred that results of proposed GA-SQP algorithm is much superior from all four algorithm based on SA, PS, NM and LM methods in case of each problem.

Complexity analyses are conducted based on the values of three operators, i.e., mean execution time (MET), mean generations (MGENs), mean number of function evaluations (MFEs). Complexity operator MET, MGENs, and MFEs are defined as the average time consumed for training of weights, average generations executed and average number of functions executed for the algorithms, respectively. The values of MET MGENs, and MFEs based on 100 independent runs of each technique for all six case studies are tabulated in Table 13. It is seen from the results given in tables that there is no significant difference in value of complexity operators in case of all six problems., i.e., all the variant of GA the values of MET, MGEN and MFE operator are around 2.75 ± 0.75 s(s), 198 ± 2 and 6000 ± 30, respectively, while the memetic versions, the respective values three operators are around 25 ± 10 s(s), 600 ± 100 and 30,000 ± 9000. The shortest time taken from GA variants in GA-6, while in case of hybrid computing approach GA-SQP-12 take smallest time for learning of weights. All the calculations for the simulation studies are conducted on a Dell Inspiron mini laptop with a 1.33 GHz processor, 1.00 GB RAM, and running MATLAB version 2012b in Microsoft Windows XP environment.

**Table 13:** Complexity analyses bases on values of MET, MGen and MFE indices

Index	Methods	Problem-1		Problem-2		Problem-3		Problem-4		Problem-5		Problem-6	
		Mean	STD	Mean	STD	Mean	STD	Mean	STD	Mean	STD	Mean	STD
MET	GA-SQP-1	40.38	8.17	14.78	1.78	29.62	7.20	16.02	10.40	11.95	4.36	15.06	2.88
	GA-SQP-2	37.56	9.12	13.93	2.01	28.05	6.62	15.47	10.40	11.57	5.08	13.91	3.41
	GA-SQP-3	38.85	6.90	14.64	1.78	30.29	7.34	19.16	10.91	11.90	3.75	14.55	3.28
	GA-SQP-4	36.41	8.30	13.39	1.40	28.78	7.20	21.22	11.01	10.82	3.25	14.01	3.34
	GA-SQP-5	39.46	7.29	14.51	1.47	29.47	7.96	17.43	11.33	11.95	2.78	14.93	3.41
	GA-SQP-6	38.76	8.04	13.69	1.39	29.53	7.82	13.49	9.75	11.42	3.98	14.18	3.35
	GA-SQP-7	37.73	8.70	14.15	1.24	29.91	7.40	19.66	11.45	11.65	3.57	14.05	4.05
	GA-SQP-8	37.83	6.20	13.65	1.37	29.01	7.55	20.86	10.75	10.81	2.33	13.56	3.91
	GA-SQP-9	38.22	8.21	14.40	1.30	29.91	6.22	18.27	10.80	11.71	1.93	14.61	3.68
	GA-SQP-10	37.19	7.97	13.58	1.43	27.72	7.11	15.66	10.26	10.72	1.46	14.24	2.90
	GA-SQP-11	38.78	7.20	14.24	1.50	30.17	6.69	19.79	10.52	11.53	1.39	13.92	4.20
	GA-SQP-12	37.36	8.11	13.41	1.10	27.44	7.77	20.20	10.01	10.52	1.77	13.86	4.10
MGen	GA-SQP-1	700.00	0.00	700.00	0.00	700.00	0.00	524.91	226.38	678.62	87.58	690.43	67.33
	GA-SQP-2	688.02	55.52	700.00	0.00	700.00	0.00	545.94	216.72	675.43	91.49	681.12	92.96
	GA-SQP-3	700.00	0.00	700.00	0.00	700.00	0.00	569.84	205.48	692.09	46.80	685.70	81.72
	GA-SQP-4	689.25	60.24	700.00	0.00	700.00	0.00	600.45	196.65	684.31	77.29	685.67	81.89
	GA-SQP-5	700.00	0.00	700.00	0.00	700.00	0.00	531.36	225.23	687.77	61.83	685.60	82.30
	GA-SQP-6	698.12	14.82	700.00	0.00	700.00	0.00	520.02	219.69	692.68	46.07	681.01	93.51
	GA-SQP-7	692.11	47.15	700.00	0.00	700.00	0.00	573.73	207.36	690.56	57.97	662.04	129.39
	GA-SQP-8	696.99	21.62	700.00	0.00	700.00	0.00	601.21	199.05	690.94	53.13	666.38	123.16
	GA-SQP-9	691.12	55.98	700.00	0.00	700.00	0.00	572.49	203.69	691.35	51.01	671.41	113.74
	GA-SQP-10	689.80	56.05	700.00	0.00	700.00	0.00	562.31	204.86	680.02	81.30	690.43	67.33
	GA-SQP-11	693.74	32.13	700.00	0.00	700.00	0.00	584.84	198.30	694.01	44.06	657.35	136.31
	GA-SQP-12	693.01	46.41	700.00	0.00	695.42	45.80	616.24	181.66	683.73	74.37	671.48	113.46
MFE	GA-SQP-1	39,020.3	4583.9	19,219.8	735.9	28,524.5	5397.6	524.9	226.4	16,724.1	4258.6	17,196.8	2364.9
	GA-SQP-2	38,348.1	6811.0	19,294.1	664.4	27,864.8	5441.3	545.9	216.7	17,137.5	4800.8	16,761.1	2743.6
	GA-SQP-3	39,021.7	4804.0	19,289.2	665.3	29,239.5	5831.6	569.8	205.5	16,904.5	3431.6	16,822.9	2647.3
	GA-SQP-4	37,477.2	6766.0	19,196.5	661.6	28,629.6	5892.8	600.5	196.7	16,610.1	2962.0	17,050.3	2696.2
	GA-SQP-5	39,290.2	5371.4	19,156.5	653.5	28,136.2	6134.7	531.4	225.2	16,757.3	2599.1	16,970.1	2803.3
	GA-SQP-6	39,251.0	5897.7	19,160.5	827.9	28,799.1	5729.5	520.0	219.7	17,034.3	3742.2	16,995.2	2846.3
	GA-SQP-7	37,769.2	6040.8	19,094.9	730.1	28,841.3	6029.7	573.7	207.4	16,724.7	3446.3	16,334.1	3528.9
	GA-SQP-8	38,841.6	4595.0	19,286.5	690.5	28,691.3	5951.9	601.2	199.1	16,677.9	2319.0	16,618.2	3435.4
	GA-SQP-9	38,365.0	6084.0	19,193.7	663.1	28,931.8	5356.7	572.5	203.7	16,625.9	1585.1	16,800.0	3224.3
	GA-SQP-10	38,162.1	6281.8	19,169.2	808.3	27,466.8	5276.7	562.3	204.9	16,413.2	1426.7	17,200.7	2398.4
	GA-SQP-11	39,193.2	5735.7	19,145.4	700.8	29,093.3	5505.6	584.8	198.3	16,541.6	924.1	16,258.2	3736.0
	GA-SQP-12	38,164.5	6395.4	19,270.1	751.2	27,210.9	6193.7	616.2	181.7	16,354.8	1774.4	16,844.8	3260.3

To elaborate the performance of proposed schemes the analyses continues by defining the global operators based on mean fitness (MFit), truncated MFit (TMFit) global MET (GMET), global MGens (GMGens), and global MFEs (GMFEs). The term global stands for the average values calculated for a number of independent executions of algorithms. The values of global operators obtained from 100 independent runs of each algorithm are listed in Table 14 and these values are based on the overall performance of algorithms in case of all six problems. Small variations in the results are generally observed, however, on the basis of MFit values the performance of GA-1 is the best, while in memetic versions, the performance of GA-SQP-10 is superior from the rest. The values of global complexity operators GMET, GMGens and GMFEs are the best for GA-QSP-12, GA-SQP-06, and GA-QSP-01 respectively. The complexity of the memetic computing approaches is a bit more than that of GAs variants but this aspect can be overshadowed due to their dominance of the performance in terms of accuracy and convergence.

**Table 14:** Comparative studies bases on values of global performance indices

Methods	MFit		TMFit		GMET		GMGens		GMFEs	
	Values	STD	Values	STD	Values	STD	Values	STD	Values	STD
GA-1	5.59E−03	9.65E−03	7.55E−04	1.42E−03	3.18	0.20	199.86	0.34	5058.20	2380.43
GA-2	4.89E−03	6.52E−03	1.37E−03	2.29E−03	2.31	0.19	199.95	0.13	5058.28	2380.22
GA-3	8.31E−03	1.29E−02	1.85E−03	1.91E−03	2.91	0.18	200.00	0.00	5058.33	2380.09
GA-4	8.75E−03	1.39E−02	1.94E−03	2.65E−03	2.13	0.18	199.62	0.65	5054.67	2379.08
GA-5	4.38E−03	6.83E−03	6.95E−04	1.30E−03	3.16	0.17	199.82	0.44	5058.16	2380.52
GA-6	4.38E−03	5.55E−03	1.41E−03	2.34E−03	2.37	0.18	199.97	0.08	5058.30	2380.17
GA-7	8.18E−03	1.33E−02	1.53E−03	1.97E−03	2.98	0.17	199.98	0.04	5058.32	2380.13
GA-8	8.81E−03	1.36E−02	2.31E−03	3.21E−03	2.19	0.18	199.51	1.19	5057.85	2381.28
GA-9	6.36E−03	1.11E−02	7.73E−04	1.39E−03	3.11	0.18	200.00	0.00	5058.33	2380.09
GA-10	7.99E−03	1.30E−02	1.54E−03	2.37E−03	2.32	0.18	200.00	0.00	5058.33	2380.09
GA-11	1.36E−02	2.44E−02	2.68E−03	2.46E−03	2.93	0.19	200.00	0.00	5058.33	2380.09
GA-12	1.23E−02	2.13E−02	2.62E−03	3.16E−03	2.14	0.19	199.38	0.84	5045.19	2374.33
GA-SQP-1	3.08E−03	6.02E−03	5.11E−09	1.25E−08	18.12	11.06	465.80	69.13	15,143.54	11,238.70
GA-SQP-2	2.04E−03	3.48E−03	6.92E−09	1.69E−08	17.77	10.38	465.14	59.07	14,933.64	10,933.65
GA-SQP-3	1.15E−03	2.54E−03	1.04E−09	2.54E−09	18.65	10.68	474.61	51.65	15,249.61	11,335.91
GA-SQP-4	2.20E−03	5.11E−03	1.40E−09	3.44E−09	18.65	10.03	476.99	37.35	14,872.66	10,776.58
GA-SQP-5	2.01E−03	3.46E−03	6.83E−09	1.67E−08	18.14	10.82	467.63	66.56	15,082.10	11,289.61
GA-SQP-6	1.78E−03	2.83E−03	7.01E−09	1.72E−08	17.80	11.24	465.34	71.46	15,235.05	11,342.57
GA-SQP-7	1.72E−03	3.37E−03	1.96E−09	4.79E−09	18.21	10.41	469.76	49.04	14,831.32	10,948.08
GA-SQP-8	2.20E−03	4.21E−03	5.82E−09	1.42E−08	18.76	10.55	476.41	37.61	15,061.59	11,228.61
GA-SQP-9	3.86E−03	7.58E−03	5.66E−09	1.39E−08	18.08	10.52	471.06	49.41	15,023.15	11,113.62
GA-SQP-10	3.24E−03	6.05E−03	8.23E−09	2.02E−08	17.53	10.32	470.43	53.49	14,770.72	10,847.38
GA-SQP-11	2.29E−03	5.08E−03	1.46E−09	3.59E−09	18.48	10.82	471.66	45.47	15,077.75	11,445.12
GA-SQP-12	2.53E−03	4.86E−03	7.25E−09	1.78E−08	18.33	10.23	477.27	31.12	14,698.36	10,850.71

## Conclusions and future research directions

Conclusions are listed as follows:

- Design of stochastic computational intelligence algorithms based on memetic computing using variants of GAs hybrid with SQP algorithm are developed in this study for solving systems of nonlinear equations arising in arithmetic benchmark, chemical equilibrium, neurophysiology, combustion theory, and economics models and proposed results established the accuracy, reliability and effectiveness.
- Accuracy and convergence of the memetic computing GA-SQP are found better than that of GAs in case of simulation studies performed for all six problems.
- Validation and verification of the performance of memetic algorithms are evaluated on the basis of statistical indicators in terms of the mean and standard deviations; which are calculated for 100 independent runs for all six nonlinear systems and small values of these indices show that proposed algorithms are consistent.
- The correctness of the proposed schemes are examined further based on values of global performance indices, i.e., MFit, GMET, GMGens and GMFEs, and results show that all given schemes provide viable but the performance of GA-SQP-4 is relatively better from the rest.
- Computational complexity analyses of the proposed design scheme GA-SQP in terms of MET, MGens and MFEs values show that there is no prominent variation found in complexity operators. However, the complexity of the memetic computing approaches is on the higher side as compared with GAs but this factor can be over-shadowed due to the superior performance of hybrid algorithms from the rest.
- Beside the provision of reliable and viable solutions of nonlinear systems of equations other valuable advantages of the proposed schemes are simplicity of the approach, easily understandable methodologies, implementation ease, readily extendable to different applications and availability of the solutions without prior known initial bias guess.

Future research opening bases on present study are listed below:

- Modern optimization solvers may play their significant role to enhance accuracy and convergence in solving nonlinear system of equations. Few recently introduced such schemes include fractional particle swarm optimization (PSO) algorithm, fractional Darwinian PSO, chaotic PSO, genetic programming, differential evolution, chaos optimization algorithms and gravitational search algorithms etc.
- One should explore to extend the application of these variants of memetic algorithms to solve stiff nonlinear differential equation, differential–algebraic systems and integral equations by transforming into a system of nonlinear equations with the help of discretization process.
